# The Bone Cartilage Interface and Osteoarthritis

**DOI:** 10.1007/s00223-021-00866-9

**Published:** 2021-06-04

**Authors:** Alan Boyde

**Affiliations:** Dental Physical Sciences Imaging Unit, Barts and The London School of Medicine and Dentistry, Queen Mary University of London, Mile End Campus, London, E1 4NS UK

**Keywords:** Bone, Joints, Articular calcified cartilage, High-density mineral infill and protrusions, Microscopy

## Abstract

This review describes results obtained with tissue from prior studies of equine and human osteoarthritis (OA). The main methods considered are scanning electron microscopy, novel methods in light microscopy and X-ray Micro-tomography. The same samples have been re-utilised in several ways. The tissues described are hyaline articular cartilage (HAC; or substitutes), with its deep layer, articular calcified cartilage (ACC), whose deep surface is resorbed in cutting cone events to allow the deposition of subchondral bone (SCB). Multiple tidemarks are normal. Turnover at the osteochondral (ACC-HAC-SCB) junction is downregulated by overload exercise, conversely, during rest periods. Consequent lack of support predisposes to microfracture of the ACC-SCB plate, in the resorption-related repair phase of which the plate is further undermined to form sink holes. The following characteristics contribute to the OA scenario: penetrating resorption canals and local loss of ACC; cracking of ACC and SCB; sealing of cracks with High-Density Mineral Infill (HDMI); extrusion of HDMI into HAC to form High-Density Mineral Protrusions (HDMP) in HAC which may fragment and contribute to its destruction; SCB marrow space infilling and densification with (at first) woven bone; disruption, fibrillation and loss of HAC; eburnation; repair with abnormal tissues including fibrocartilage and woven bone; attachment of Sharpey fibres to SCB trabeculae and adipocyte-moulded extensions to trabeculae (excrescences).

**Supplementary Information:**

The online version contains supplementary material available at 10.1007/s00223-021-00866-9.

## Introduction

Osteoarthritis is generally recognised as a disease of all the tissues in and around a joint, but the commentary here is confined to what is learnt by studying cartilage and adjacent bone in synovial joints—not the synovium and synovial fluid—by novel microscopic methodologies. The subject matter concerns OA. We should be able to document a progression from normal, but to do that we should know and show what is normal. We cannot have human material to study early stages of development, but we have ample equine material. There are many who believe that rat and mouse studies are relevant, but it is debatable whether the joint loading is comparable. Nevertheless, mouse material permits the rapid investigation of possible genetic factors.

Different insights are given by novel investigative methods applied to old problems, in the present case, the changing structure of the osteochondral junction region from the normal mature individual until the development of age changes and osteoarthritis (OA). Tissue studied was derived from prior studies of equine and human OA. The methods considered here are preparative methods for samples for scanning electron microscopic (SEM) study; imaging techniques in SEM; novel methods for preparing samples which can be used for both conventional light microscopic (LM) study and SEM—Laser Ablation Microtomy—and correlation with X-ray Micro-tomography (XMT, µCT) and novel LM methods. The same samples can be re-utilised in several ways. For PMMA embedded-tissue blocks, for example, we first examine them after micromilling or polishing by backscattered electron (BSE) SEM to study variations in mineral concentration and then stain them with iodine to view the general histology of un-mineralized matrix and cells. Then we remove *either* the calcified parts to study the space compartments (bone marrow, blood vessel canals) *or* plasma ash to remove the PMMA and study the mineralized parts. The last aim can be achieved directly by wet, ‘macerating’ methods. The tissues described are hyaline articular cartilage (HAC; or substitutes), its deep layer, articular calcified cartilage (ACC), whose deep surface is resorbed in cutting cone events to allow the deposition of subchondral bone (SCB). Multiple tidemarks are normal. Turnover at the osteochondral (ACC-HAC-SCB) junction is downregulated by overload exercise, conversely during rest periods. Consequent lack of support predisposes to microfracture of the ACC-SCB plate, in the resorption-related repair phase of which the plate is further undermined to form sink holes. The following characters contribute to the OA scenario: penetrating resorption canals and local loss of ACC; cracking of ACC and SCB; sealing of cracks with High-Density Mineral Infill (HDMI); extrusion of HDMI into HAC to form High-Density Mineral Protrusions (HDMP) in HAC which may fragment and contribute to its destruction; SCB marrow space infilling and densification with (at first) woven bone; disruption, fibrillation, and loss of HAC; eburnation; repair with abnormal tissues including fibrocartilage and woven bone; attachment of Sharpey fibres to SCB trabeculae and adipocyte-moulded extensions to trabeculae (excrescences).

## Materials

This review considers material from prior equine and human studies, which was all obtained with full ethical permissions required at the time and as per the relevant referenced publications.

### Equine

A range of third metacarpal bones (Mc3) from TB racehorses obtained from birth to 24 years [1]. This included ‘aged’ or evidently pathological material retrieved from racing and training fatalities, especially fetlock joints.

Right Mc3s from twelve 2 year old female TBs [2] from a treadmill training experiment organised by Allen Goodship and Helen Birch at Bristol in 1996 [3, 4].

New Zealand TB foals, material collected by Elwyn C Firth when at Palmerston North.

MUGES: 14 two-year-old TB fillies reared entirely at pasture: seven underwent training: the other 7 fillies were confined to large grass paddocks and not trained [5–13].

GEXA, PASTEX, CONDEX; distal Mc3s from 33 TB foals divided into exercise and control groups as described in [14–16].

TB racehorse hock joints (Bathe et al., unpublished).

The Hong Kong TB POD study: [17–20].

Icelandic horse tarsal joints (Ley et al.) [21, 22].

Standardbred carpals (Laverty and collaborators) [23].

### Human

A collection of OA femoral heads retrieved at arthroplasty or *post mortem* controls (HOACC, Oswestry, Chris Sharp [24]).

Femoral heads removed at arthroplasty from OA and AKU-OA cases from Gallagher and Ranganath and collaborators, Liverpool [25–29]

Proximal tibia and distal femur removed at arthroplasty from OA cases, Nidhi Sofat, SGUL [30, 31]).

## Methods

Most of what is described derives from special microscopic methods and inter-method correlations which were initiated to study bone and joint tissues [32–34].

### Scanning Electron Microscopy (SEM)

SEM is not a single method. There are several branches of SEM in bone and joint research, each with their own advantages and they can be combined in different ways [35–44].

*First,* to look at 3D matrix and mineralizing front preparations. In bygone years, this mostly involved pre-preparation (‘maceration’) to remove all cells from matrix surfaces or unmineralized matrix from mineralizing fronts, i.e. making an anorganic or deproteinized sample, but importantly, older SEMs required the addition of a surface conductive coating to give electrical ground potential under relatively high (‘good’) vacuum. Imaging was done with low energy secondary electrons (SE), and to increase this yield we commonly employed high atomic number metal coatings (Au, Au–Pd, Ag) by sputtering or evaporation. This is no longer necessary with modern instrumentation: by operating at a higher pressure (worse vacuum) we achieve charge neutralisation by attracting positive gas ions to the erstwhile negatively charged surface. This does not improve resolution, but it is a revolution in convenience and specimen turn-round time. However, large and complex they be, we no longer need to coat samples and wait for long periods to get a ‘good’ vacuum.

*Second,* we use high energy backscattered electron (BSE) detectors either for compositional, atomic number contrast imaging to study mineralization density or for 3D topographic imaging. If we wish to concentrate on the former, we have to eliminate—or at least to reduce—the influence of the latter: i.e. the sample must be flat [35–39]. For it to be flat and free of voids, it has first to be embedded. In our own hands, we have always preferred (poly)-methyl-methacrylate (PMMA) for this purpose because it can be micro-milled or polished to give minimal surface relief (epoxy resins are notoriously difficult to polish) [44].

*Third,* we exploit PMMA blocks by iodine staining their surfaces, which can be done dry using iodine vapour [44–46]. We now have a very large library of beautiful images of joint histology obtained with this approach. The advantages are that there is virtually no limit to sample size [other than in thickness, or should we say thinness of the tissue sample to get the MMA monomer to penetrate in the first instance] and that the image derives from a one half to one micron thick layer in the block surface. The PMMA holds all the tissues together: there is no decalcification step: unstained mineral-content-dependent imaging can be done first etc.

We may also embed tissue in an iodinated methacrylate in the first instance, whereby we achieve a ‘negative staining’ contrast.

*Fourth*, we look not at the sample itself but a replica, with either SE or BSE 3D imaging. The replica may be an internal *cast*, made by dissolving away the mineralized tissues from PMMA embedded material to show the fine space compartment occupied by ‘lacunae and canaliculi’ or—and usually much more usefully—the larger gauge blood vessel canals and marrow space in bone after dispersing the former [38, 44]. It may alternatively be a one or a two stage *replica* of a surface—and that surface can be a wet surface—which is hugely advantageous in studying joint cartilage surfaces [47], but yet to be applied wholesale to large joints such as those of man and horse.

*Fifth*, we may remove the embedding resin and any non-mineralized matrix from a polished sample to gain 3D information about the mineral front by plasma ashing.

*Sixth*, we mention cross-correlation with non SEM methods including light microscopy (LM) and X-ray methods. The PMMA block surface is perfect for confocal scanning LM (CSLM) [8–10, 43, 44]. All blocks and slabs can be imaged with Faxitron point projection X-ray microscopy. The true block surface will be discovered by X-ray microtomography (XMT, µCT) lending to exact correlation with SEM and CSLM.

### SEM Instruments

From 1967 to 1992, we used Cambridge Instrument Company Stereoscan S1 and S410 SEMs. From 1992 to 2011, all imaging in our labs (both at UCL and QMUL) was done using a Zeiss DSM 962 automated digital SEM (Zeiss UK, Welwyn Garden City, Herts UK) with an annular solid state BSE detector KE Electronics Ltd, Toft, Cambs UK), 20 kV. However, since 2011 we have used a Zeiss EVO-10 SEM with vapour pressure control in the sample chamber, when the specimens may be examined uncoated at, for example, 50 Pa chamber pressure. As noted above, the electron beam ionises the residual gas in the chamber and positive ions neutralise the negative charge in the sample.

### Backscattered Electron Imaging of PMMA Embedded Bone Samples

BSE-SEM is the most important method of studying the density distribution in mineralized tissues at high resolution [35]. To quantitate mineral content we used halogenated dimethyl methacrylate standards against which to calibrate electron back scattering coefficient [39].

### Light Microscopy, CSLM, PLM, LAM

Although we have the general tendency to eschew classical LM methods because they remove the hallmark mineral component, decalcification obviously makes it much easier to cut sections to which a bewildering array of general and immune-specific staining methods can be applied. Mechanically microtomed sections of undemineralized tissues and whatever embedding material are a morphological nightmare and I take the liberty to emphasise this here because it is so incredibly and widely overlooked.

The most excellent LM sections are to be obtained with the technique of laser ablation microtomy (LAM) [48]. With LAM we have permanent preparations which can be studied with all SEM, LM and X-ray methods. Without the equipment we have had to make do with what we have got, and I shall show some examples of decalcified section histology in the context of polarised light LM (PLM).

### Dynamic Aperture Microscopy (DAM)

A unique method for producing instant three-dimensional images through a conventional light microscope creates 3D perception using motion parallax cues, rather than stereo parallax information [49]. The method employs a pie-shaped, rotating aperture mask, creating oblique illumination (or oblique viewing) of the specimen from different angles. Rotating the aperture mask continuously changes the angle from which the specimen is viewed, and the human eye/brain complex converts the motion parallax into a three-dimensional perception. 3D confocal-like images can be generated from conventionally stained specimens using standard bright field optics. This is, of course, dependent on video-imaging and cannot be reproduced on the printed page.

### New PLM Method

We have developed new methods for extending the usefulness of PLM in bone and cartilage studies. The driving motivation was to exploit the high quality, thin, undecalcified sections produced by LAM which enable exact correlation with backscattered electron scanning electron microscopy imaging. We exploit digital image processing to pack more useful 3D information into an image. We constructed a PLM in which the polarising and analysing filters are—under computer control—rotated together for multiple rotations at usually 7.5° or 15° intervals through a range of 90° with monochrome images recorded at each position. It will be simplest to describe the processing of 6 images at 15° rotation intervals: we can start with any image, but we combine them in the colour circular sequence Red, Yellow, Green, Cyan, Blue, Magenta. Colour in the composite image shows the (mainly collagen) orientation within the section plane. Brightness is proportional to the cosine of the strike angle with respect to section plane, being brightest when the collagen is in plane, and black when perpendicular to that plane, i.e. parallel to the optic axis. Summing the set of images produces results which are equivalent to using circularly polarised light [50, 51].

## Results

**Fig. 1:**
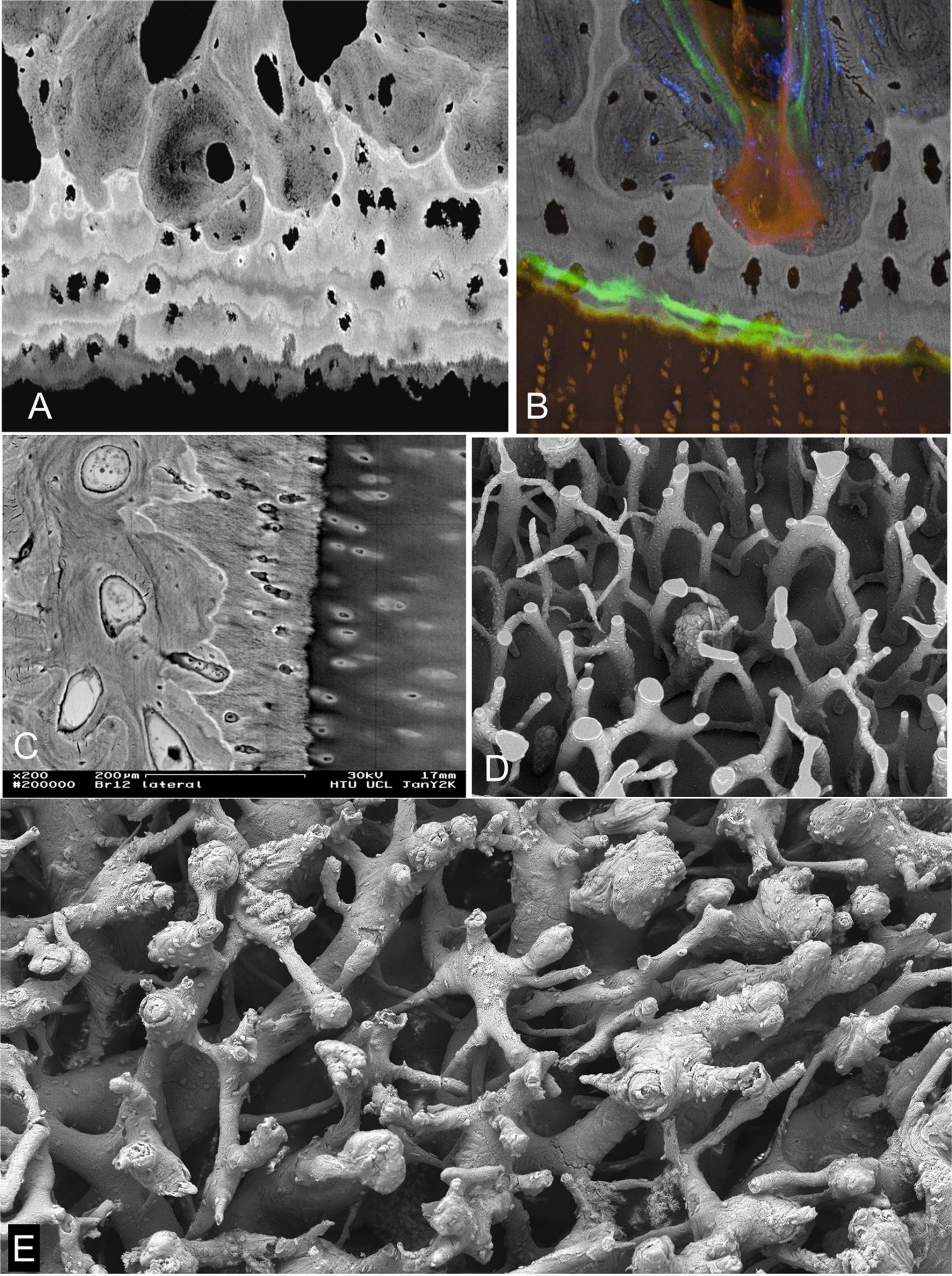
Normal osteochondral junction. **a** 20 kV BSE-SEM image of carbon coated, micro-milled PMMA embedded block surface of distal condyle of 2 yearr old Thoroughbred (TB) racehorse third metacarpal bone (Mc3). HAC (at bottom) hyaline articular cartilage region which here shows black as it contains no mineral. ACC shows incremental mineralization-front-progress layers with more and less mineral content. Much of the ACC is more highly mineralized than the subchondral bone (SCB, top): SCB is attached—via a hypermineralized (reversal) cement line to the deep layer of the ACC after local ‘cutting cone’ resorption events. FW = 594 µm. **b** 20 kV BSE-SEM of 2 year old TB Mc3, embedded in PMMA and carbon coated, combined with confocal fluorescence image stack of same field, showing brown yellow autofluorescence in HAC and marrow space in SCB and green fluorescence of two calcein mineralizing front labels in both SCB and ACC. Micrograph by Michael Doube. FW = 500 µm. **c** 30 kV BSE-SEM of 2 year old TB Mc3 embedded in IEMA iodinated methacrylate resin, carbon coated. The embedding resin has replaced water in the original tissue structure_._ Here the iodine acts as a negative stain to contribute additional BSE signal to that due to the mineral component proper. FW = 580 µm. **d** 20 kV BSE-SEM image of gold coated PMMA cast of mid-shaft transverse section of 2 yr old TB Mc3, made by dissolving embedded bone with sequential acid and bleach treatments and ultrasonic dispersal of osteocyte lacunar and canalicular casts. Most of the architecture shows the primary osteonal canal system, but near the centre can be seen a ‘cutting cone’ coming towards the observer, with detail of the individual resorption pits made by the teams of osteoclasts. FW = 1937 µm. **e** 20 kV BSE-SEM image of gold coated PMMA cast of marrow space at the osteochondral junction in a 2 year old TB Mc3 condyle, made by dissolving embedded bone with sequential acid and bleach treatments and ultrasonic dispersal of osteocyte lacunar and canalicular casts. Many ‘cutting cone’ replicas are seen, large ones with detail of the individual resorption pits by osteoclasts contrast with narrow projections which show the space in shut-down ‘osteones’. [A version in which colour shows direction of collection of the BSE signal [40, 41] will be found in the supplementary figures]. FW = 1900 µm

All ACC shows an incremental stratification in mineralization density very well seen in BSE-SEM images of micro-milled or polished PMMA block surfaces (Fig. 1a–c). That the layers represent differences in the degree of mineralization which occurred at successive time intervals seems intuitively obvious, but it is also proven by the visualisation of time given by tetracycline and calcein labels seen with CSLM imaging overlaid on to the BSE imagery (Fig. 1c) [8–10].

HAC contains no mineral and with normal set-up conditions for BSE-SEM appears ‘black’ (Fig. 1a). In fact, the organic matrix of cartilage does not have an identical electron backscattering coefficient to that of PMMA and it is possible to use gain and contrast values to see detail in the matrix, but under those conditions it is difficult to see the variations in mineral content which make the ACC such a fascinating material. By using non-linear gamma corrections and local contrast enhancement filters it can be possible to see detail in both HAC and ACC in the same image, but staining achieves this object more simply and reliably.

Negative staining during embedding by using an iodinated monomer IEMA co-polymerised with MMA effectively demonstrates what was the water content in cartilage matrix, since this is replaced with the resin. This is not uniform, as shown in Fig. 1c.

Positive staining of embedded block surfaces with iodine stains everything, including mineralized bone and cartilage, but the greatest transformation occurs in osteoid and cartilage matrix such that cells and fibre orientations and general ‘histology are easily visualised Quick staining is done with a solution of iodine in ammonium iodide applied for say 30 min: it is necessary to wash off excess stain which may puddle into deficiencies in the block surface (Fig. 8e). Slow but very reliable staining is done with iodine vapour (Figs. 7e, 8c, 8d) [21, 22, 44–46].

A major concern in understanding the norm and the progression to OA concerns the remodelling and turnover activity in the depths of the osteochondral junction beneath the HAC. Here cutting cones penetrate from SCB into ACC and re-establish and renovate the junction between these two tissues, fundamental to the maintenance of the attachment of the HAC to the bone organ.

In our studies of equine bone we were presented with the opportunity to examine this activity in relation to the exercise status of the whole animal and whole bone organ. In particular, in the Bristol 1996 treadmill [2] and the Massey University Grass Exercise Study (MUGES) training experiments [6] we could compare identical regions of animals of the same age which had undergone extra training exercise over and above baseline exercise. In the course of such studies we noted that there might be a difference in the number of cutting cone events between the control and exercise groups. However, in studying the single electron optical section available in the PMMA block surface—essentially a half micron thick section as extensive as the block itself—we could observe only a tiny fraction of cutting cone activity that might have been present in the block.

Methods were progressively investigated and perfected, using tissues from horses whose response to exercise had already been described [5, 6] to obtain suitable preparations that would allow us to determine if the features of cutting canals in the SCB/ACC tissues were different in exercise-trained and control horses.

By ‘casting the osteochondral junction’, we exploited existing PMMA embedded material by converting the blocks into casts of the principal space compartments of the calcified tissues by dissolving the latter away completely. A main difficulty with this technique is that bone tissue proper is so full of cells that the ‘trees cannot be seen for the wood’, i.e. it appears that the bone had never been removed because the 3D continuum of osteocyte lacunae and canaliculi blocks the view of the marrow spaces as such.

To make the ‘cast’, the mineral component is dissolved by treatment with 2 N hydrochloric acid, leaving the samples for days at a time. The thus exposed organic matric components are then dissolved by treatment with strong hypochlorite bleach. The HCl and NaOCl treatments are repeated sequentially over a period of many days or weeks (Fig. 1d, e).

The resulting PMMA cast is stripped of the super-abundant, poorly attached osteocytic lacunar and canalicular components by sonication and washing with a gentle water jet. The clean cast is rinsed with distilled water and preferably freeze-dried to avoid the surface tension forces causing the cast to collapse as may happen if the cast is simply blotted and allowed to dry at room temperature. This is achieved by freezing by holding it above boiling liquid nitrogen (− 196 °C); placing in a vacuum chamber attached to a rotary pump and leaving it there until all ice in the sample has sublimed and the sample has equilibrated to room temperature.

Originally, the dry casts were given conductive coatings of carbon by evaporation whilst tilting the sample through a large range of angles to assure even carbon coverage and penetration deep into the spaces within the cast and permit examination in a high (good) vacuum. The cast was then further coated with gold by evaporation (again with tilting to assure even coating) to increase both the SE and the BSE signals arising from the very surface of the cast elements.

A technical problem for studying the osteochondral junction region in casts concerns any continuity of methacrylate from SCB marrow space via any patent canals penetrating the entire thickness of ACC through to HAC. The large water component of HAC means it comes to constitute a solid methacrylate element. Nevertheless, in the initial studies of the MUGES slices, the resulting casts, typically 1–2 mm deep, permitted us to see much further in 3D into the tissue than in the original polished PMMA surface. We soon realised that much more information could be obtained if the part of the cast relating to the HAC could be removed because this was essentially a dense block of PMMA owing to the high water content of HAC. To achieve this, we embedded macerated ‘wedges’ in which the HAC had already been removed to the level of the ACC MF, assuring the resin would penetrate from the SCB side by removing bulk epiphyseal trabecular bone deep to the dense SCB plate. The problem of penetrating canals was overcome by coating the ACC MF with alginate separation medium prior to embedding. Following the acid and bleach treatments the extraneous PMMA external to the tissue could be easily cleaved off. We are left with methacrylate casts of soft tissue spaces (marrow, blood vessel canals) within solid bone: the largest volumes represent the marrow space compartment in spongy bone. The terminal elements of the methacrylate in the immediate dense ACC and SCB bone plate domain provide meaningful images of turnover. Examined in detail, the cast usually has the layer of osteocytes closest to the marrow space attached to the marrow space cast by canalicular elements. The amount of PMMA in the general bone matrix between osteocytes and their canaliculi varies with maturity of bone tissue, being greatest in newly formed and least in the most mature domains. In newly formed, immature bone packets, this intercellular PMMA phase can cause all cell lacunae and canaliculi to be retained in the cast. The technique thus also allows visualisation and recognition of bone that has been formed more or less recently.

However, the structures of interest were not these, but rather those which had no retention of osteocytic debris, which are of two types (Fig. 1e):

A. Large dome-shaped elements with a surface structure reflecting the presence of osteoclasts within the space domain of the previous cutting cone penetrating the ACC, covered with the negatives of the osteoclastic resorption lacunae. In some A elements, where resorption had been reversed and new bone formation was starting, the resorption lacunae profiles are less visible and the first osteocytic casts are retained. B. Narrow projections generally remarkably free of attached osteocytic elements, which are the canal space of blind-ended osteonal canals, a high proportion of which had been located with ACC. The very narrowest of these indicate already shut down systems and have an obviously pointed tip.

It is easiest to understand and interpret the 3D cast data in compact bone (Fig. 1d).

We found that the numbers of new cutting cones (and osteons) in the diaphyseal bone of exercised animals was reduced, but this was also true for the level of the osteochondral junction. In animals where a higher level of exercise had been maintained until euthanasia. The preponderance of Type A protuberances, meaning recent cutting cones penetrating into ACC, was greater in rested horses and of Type B—shut-down system—in animals with high exercise levels.

**Fig. 2:**
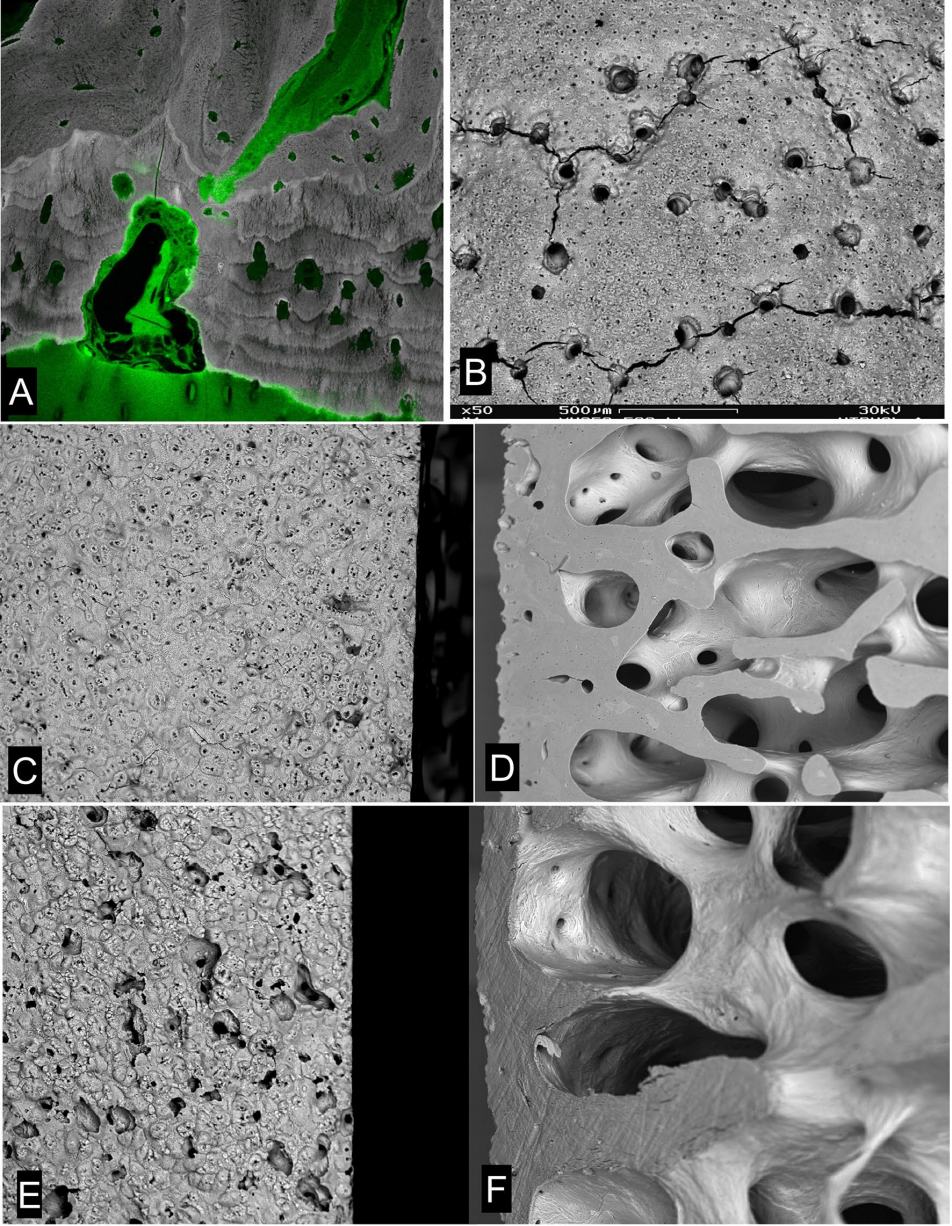
HAC invasion by cutting cones. **a** 20 kV BSE-SEM of 2 year old TB Mc3, embedded in PMMA and carbon coated, combined with confocal fluorescence image of same field, showing green autofluorescence in HAC and in marrow space in SCB. A resorption–cutting cone–event has penetrated right through the ACC and into HAC. FW = 400 µm. **b** 30 kV BSE-SEM of macerated 2 year old TB Mc3 condyle showing cluster of cartilage canals on the median sagittal ridge. Many are connected by artefactual cracks which arise during drying due to the presence of underlying blood vessel canals. FW = 2084 µm. **c**, **d** 20 kV BSE-SEMimages of vertical section through superior surface of femoral head of 33 year old male prepared by maceration with alkaline bacterial pronase at 50 °C to remove non-mineralized tissues. A normal view of the ACC MF (left) contrasts with a view of a cut and polished surface after 120 degrees rotation at right. A few sites can be seen there is a ‘canal’ (a void) connecting the SCB marrow space with the HAC (now a space because it has been digested away), which was present above the surface in C to the left in D. Field widths both 2700 µm. **e**, **f**. 20 kV BSE-SEMimages of vertical section through superior surface of femoral head of 75 year old male prepared by maceration with alkaline bacterial pronase at 50 °C to remove non-mineralized tissues. A surface normal view (**e**) contrasts with a view of the cut surface after 140 degrees rotation in F. Many sites can be seen where voids connected SCB marrow space with the HAC (which was, above the surface in E and to the left of F). Field widths both 2700 µm

Cutting cones penetrating the ACC from the bone side cause local loss of the HAC connection to the bone organ (Fig. 2). In normal young mammals, the extent of cutting cones going right through the ACC to meet the HAC is limited after initial very rapid growth periods, when it is everywhere extensive. In 2 year old TB horses we could always find patches with penetrating canals in the midline sagittal ridge of the third metacarpal and metatarsal (cannon) bones, but very few elsewhere (Fig. 2b). The presence of cartilage canals of course means that bone is not attached to joint cartilage in the usual way. In adult human femoral head material, we will find a few places where ACC is missing, but the proportion of this missing junctional tissue increases with age (Figs. 2a–d), The extent to which these voids contribute to the whole OA scenario is unknown, but such regions are very common in OA and the lack of attachment may be a contributory factor in OA.

**Fig. 3:**
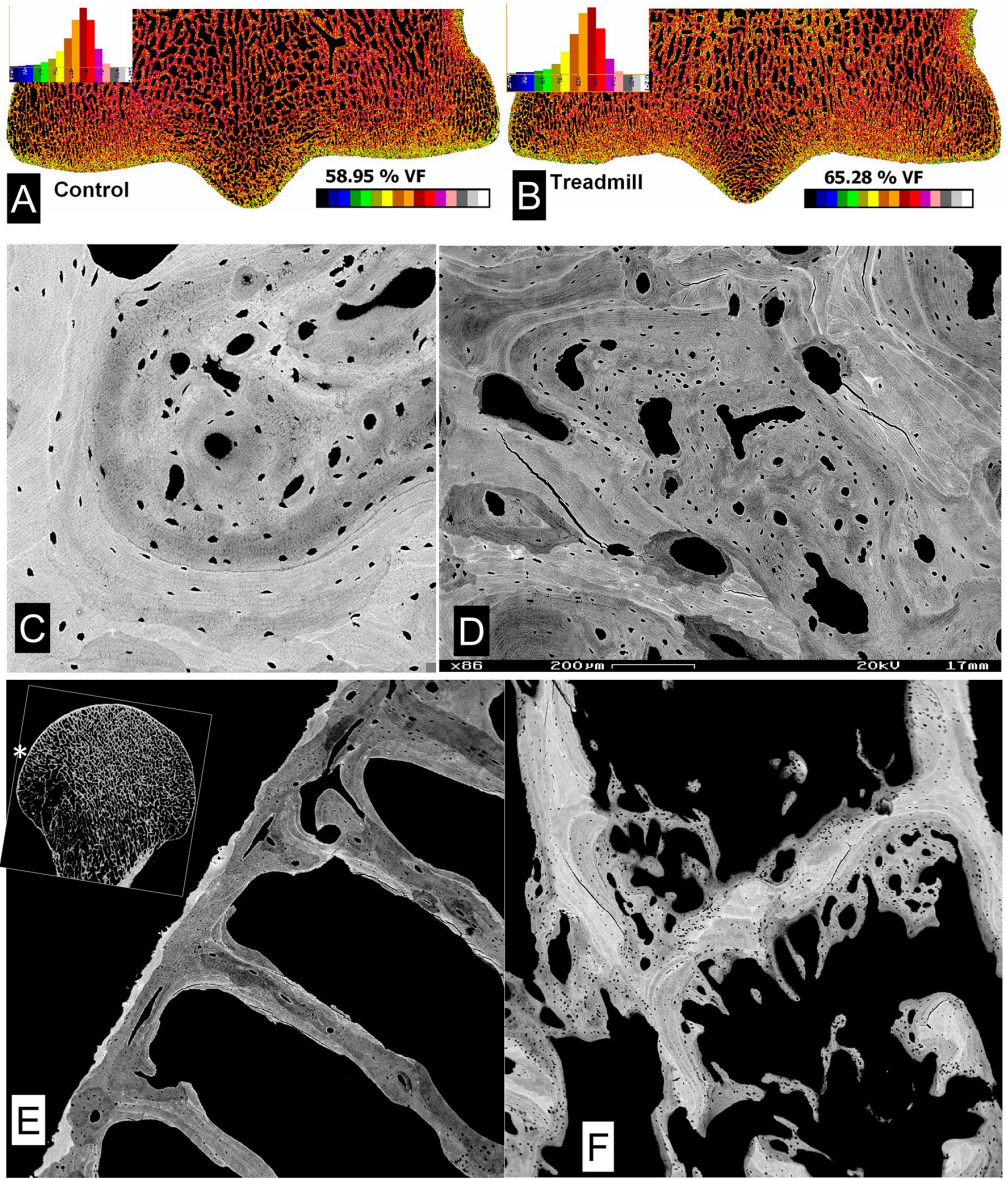
Densification by forming new bone in marrow space in response to exercise/loading. **a**, **b** Quantitative BSE-SEM image montages of medio-laterally micro-milled block faces, carbon coated, of two 2 year old TB MC3s from the Bristol 1996 training experiment which had received control A and treadmill B levels of exercise. The mean bone volume fraction increases with exercise, but the extra bone has a lower mineralization density as indicated by the shift of the image histogram to the left in B. Field widths both 53 mm. **c** 20 kV BSE-SEM2 year old TB racehorse Mc3 from treadmill trained group of the Bristol experiment. A prior marrow space is entirely filled with bone, much of which originated from the more densely mineralized woven bone strands formed near to blood vessels in the centre of this space, i.e. remote from the nearest bone surface. Other bone has been deposited on the prior cancellous bone without a resorption step, and there is no cement line. The stimulus resulting in this extra bone was transmitted via the HAC and ACC without any change which could be detected in either of these tissues. FW = 1036 µm. **d** Densification by forming new bone in prior marrow space in SCB in human OA femoral head. Quantitative BSE-SEM image. FW = 1350 µm. **e** 20 kV BSE-SEM showing part of the osteochondral junction in the femoral head of a PM reference/control case from the Oswestry study [24]. The location of the field is shown by the asterix in the inset image which shows a single XMT data slice ~ 500 µm in the block surface. The ACC is extremely thin—the narrow white strip at the left border of the tissue. Both the subchondral bone plate and the radiating trabecular plates have blood vessel canals in their centres. A pseudocoloured version of this image will be found in the supplementary data files. FW = 1782 µm. **f** 20 kV BSE-SEMshowing deep subchondral trabecular bone in an OA (elective arthroplasty) femoral head from the Oswestry study [24]. Note the many strands of woven bone in the marrow space and attached to the trabecular plates, which do not have central BV canals. Differences in the fabric density of the old and the repair bone are best seen in a pseudocoloured version of this image which will be found in the supplementary data files. FW = 1114 µm

Levels of exercise/training which reduce remodelling in compact bone and at the osteochondral junction also induce new bone formation in the subchondral domain (Fig. 3a–c). The only place and space where new bone can be formed is within the fatty marrow and this is where it forms. But there are two mechanisms involved in this densification process of increasing the bone volume fraction. *First*, new lamellar bone is added to existing trabecular/cancellous bone surfaces—but *without* any prior resorptive event. This new bone may be deposited upon a relatively *poorly—*or even on an *un-*mineralized bone surface, so that the junction is in this sense abnormal. In the process of new bone formation coupled to a prior resorptive event, we would normally find a hypermineralized ‘cement’ line (Fig. 1a). *Second*, new bone formation is initiated as immature woven bone strands remote from existing bone surfaces within the fatty bone marrow—and the circumstantial evidence from our equine training experiment data is that this may happen first, and very rapidly, within days, after a bout of vigorous exercise (Fig. 3a–c).

**Fig. 4:**
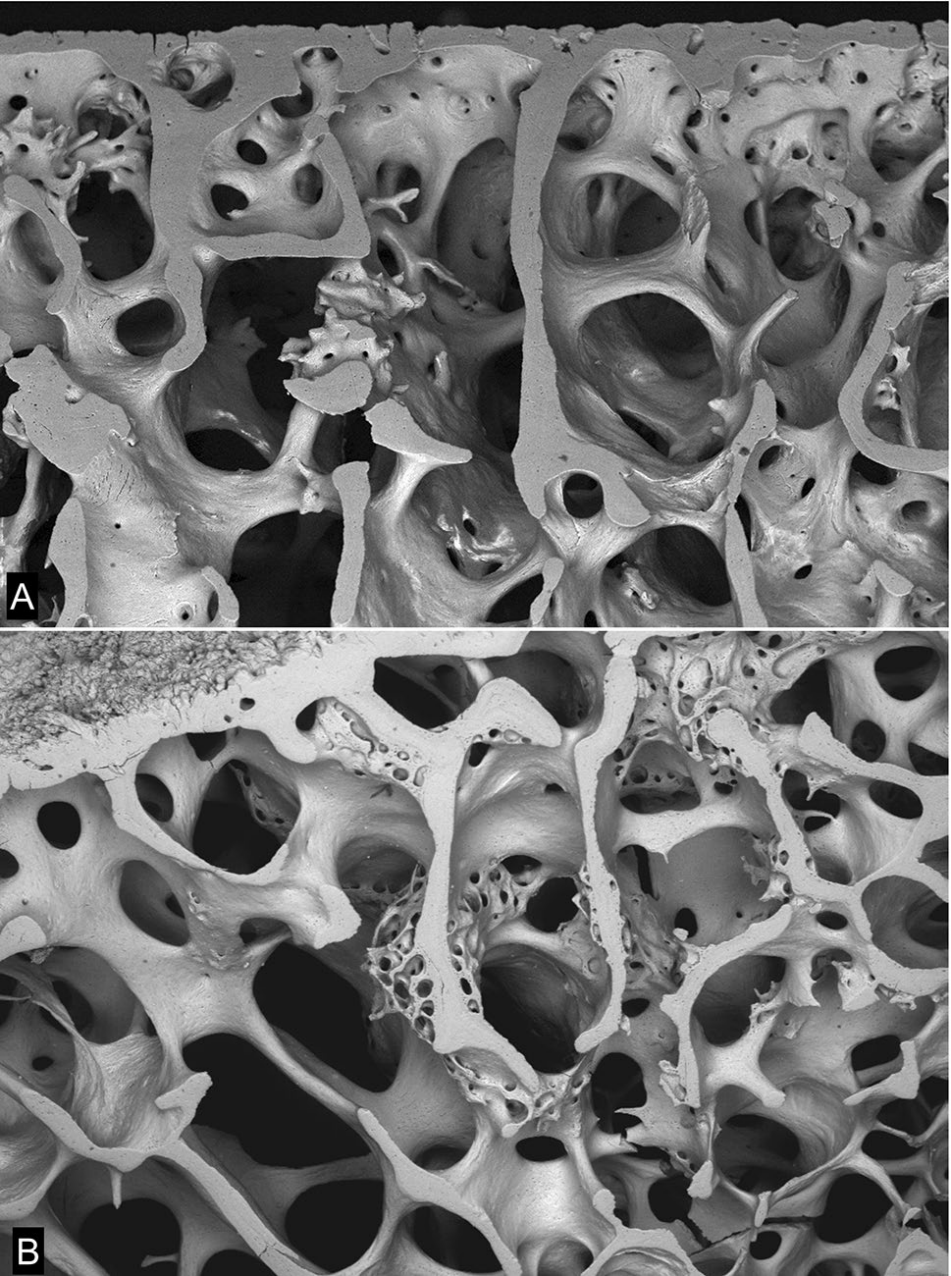
Distribution of new woven bone in prior marrow space in OA. **a** 20 kV BSE-SEM image of vertical section through superior surface of femoral head of 69 yr old male (‘normal’ PM case) prepared by maceration with alkaline bacterial pronase at 50 °C to remove non-mineralized tissues. This view is tilted to show the underside of the thin subchondral bone plate with the openings of canals which penetrate through the ACC, and woven bone callus formations which increase the volume fraction of bone. FW = 4689 µm. **b** 20 kV BSE-SEM images of vertical section through superior surface of femoral head—OA arthroplasty case prepared by maceration with alkaline bacterial pronase at 50 °C to remove non-mineralized tissues. The tilted view shows the outside of the subchondral bone plate with its covering of ACC (top left), with woven bone callus formations internally. One of a through tilt series. [Movie in supplementary information]. FW 5000 µm

This phenomenon of increasing bone volumetric density with new bone which itself has a lower mineralization density occurs without there necessarily being any change detected in either the HAC of the ACC. The load necessary to induce these changes in bone can thus be transmitted through all cartilage and without any change in the cartilage [2, 4, 6, 9–12, 52, 53]. Although we can also find instances in our equine material where changes in both HAC and ACC are present, want to emphasise that the overwhelming impression is that the evident bone changes happen first. The same types of change are seen in human OA (Figs. 3d, e, 4 and see later) but we cannot have the numbers of early-stage observations in human material that we have accrued from overload studies in younger horses.

The most reliable way of demonstrating new isolated strands and clumps of immature woven bone formed in erstwhile fatty marrow space in response to overload exercise is to study PMMA embedded bone blocks (Fig. 3f). The resin keeps everything in place, and nothing can fall out—as happens with conventional histology sections and which may also occur in macerated specimens prepared for 3D SEM.

**Fig. 5:**
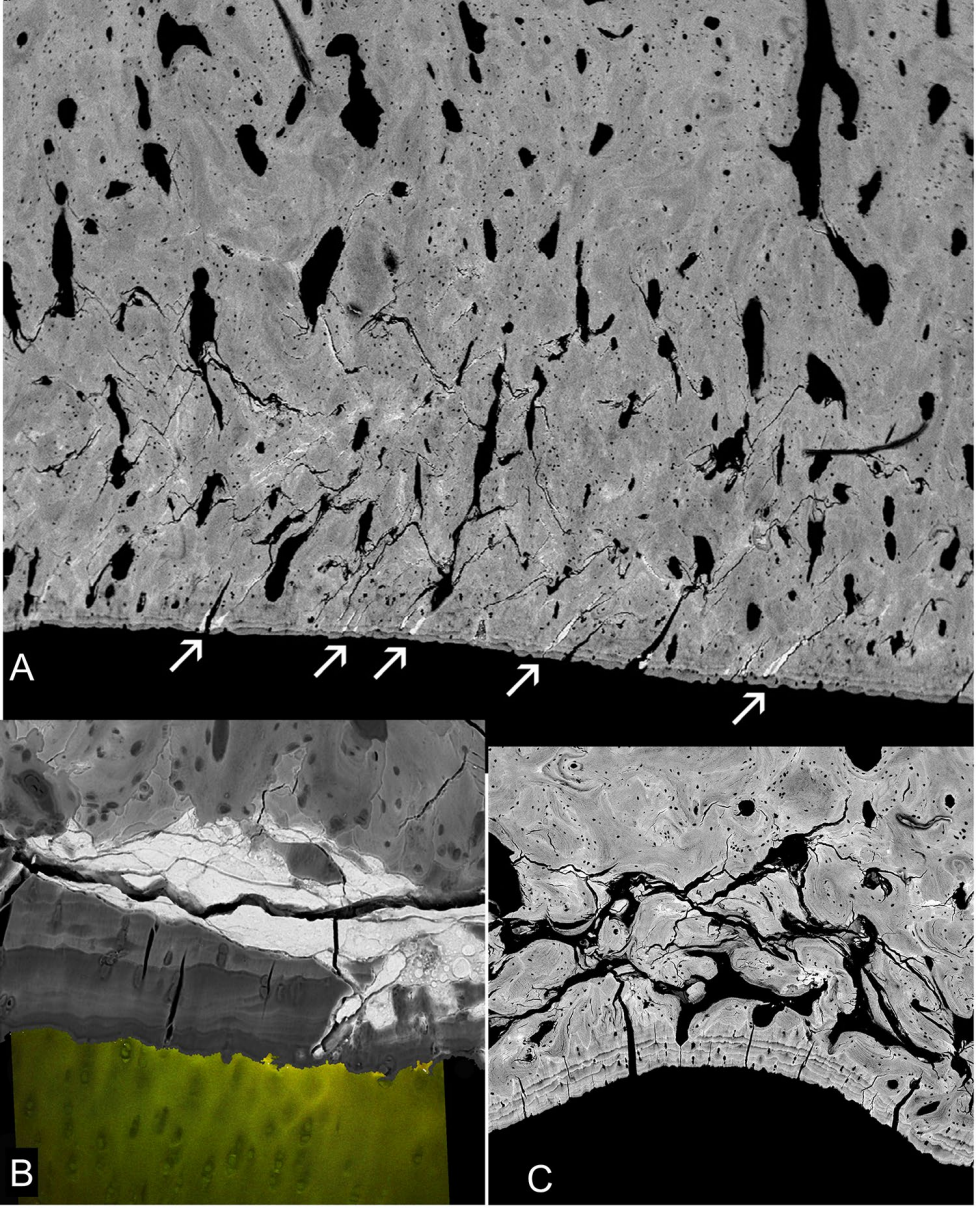
Cracks in ACC and SCB after overload exercise in TB Mc3s. **a** 20 kV BSE-SEM image of 5yo TB Mc3 condyle showing linear cracks in ACC aand extensive criss-cross cracking and compression of SCB, with crack repair with high-density mineral infill (HDMI) material. The normal MF has recovered in ACC above healed and sealed cracks at arrows. HDMI is a general crack healing, sealing, and annealing phase found in all calcified tissues. FW = 4450 µm. **b** 20 kV BSE-SEMof TB Mc3 condyle, embedded in PMMA and carbon coated (top), combined with confocal image of same field, showing yellow green autofluorescence in HAC in lower part of field. HDMI has filled an extensive crack system, but the MF of the ACC has progressed to include this phase within ACC. FW = 636 µm. **c** 20 kV BSE-SEMimage, TB Mc3 showing concave depression in palmar condyle with compression fracture in the SCB and patches of HDMI. FW = 1782 µm

**Fig. 6:**
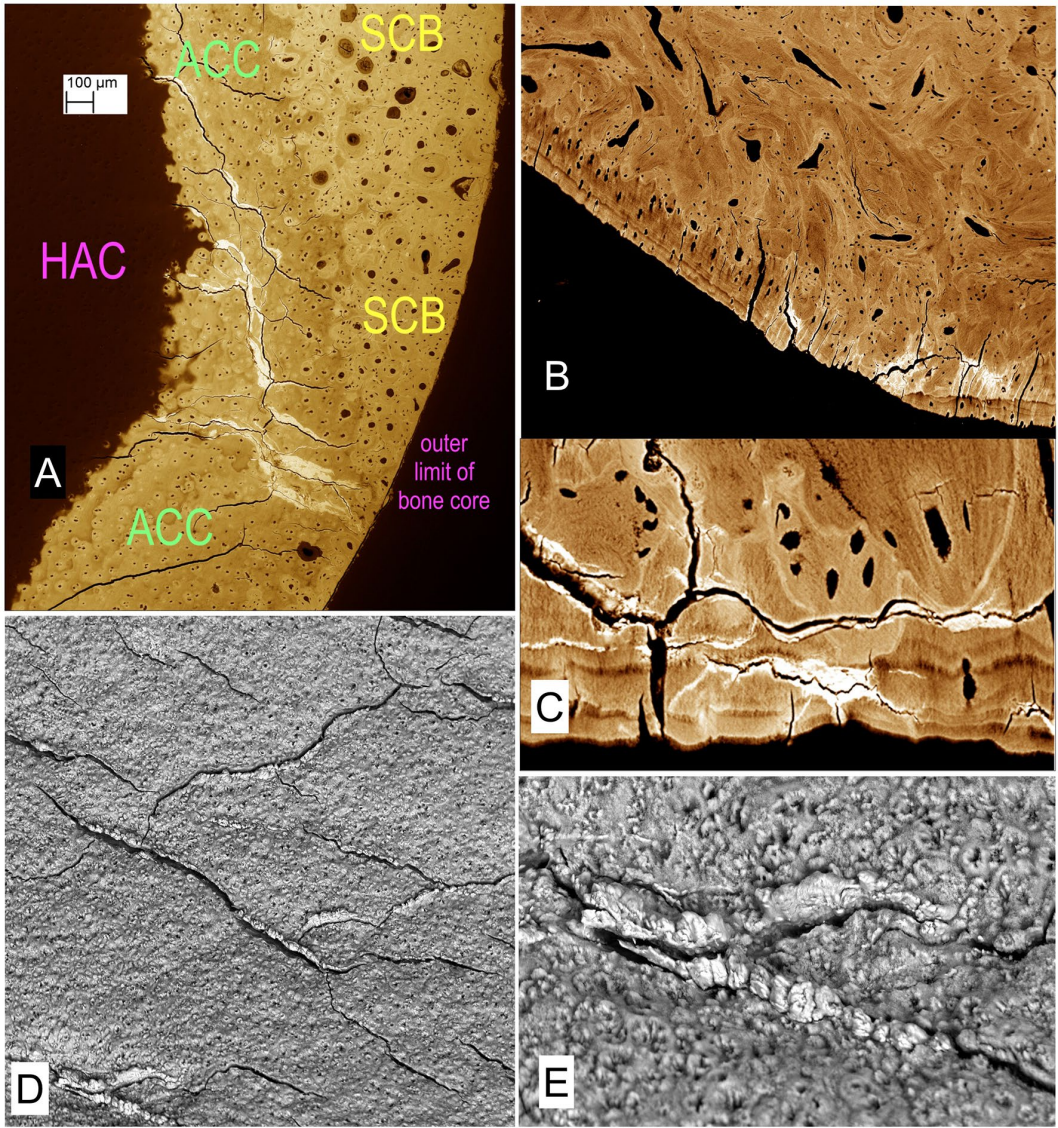
Cracks in ACC and HDMI in. Standardbred carpus and TB Mc3s. **a** 20 kV BSE-SEM of Standardbred horse carpal bone 1 cm core sample embedded in PMMA, polished surface nearly perpendicular to joint surface: showing extent of cracks in ACC filled with HDMI. Uncoated sample, chamber pressure 50 Pa. From study of naturally occurring post-traumatic equine carpal osteoarthritis in Standardbred horses with Prof Sheila Laverty, Montreal. FW = 2039 µm. **b** 20 kV BSE-SEM of TB Mc3 condyle, embedded in PMMA and carbon coated. Region with extensive HDMI in cracks in HAC towards lower right of field has been overlain by normal ACC, i.e. the MF of the ACC has recovered. FW = 1782 µm. **c** 20 kV BSE-SEM of TB Mc3 condyle, embedded in PMMA and carbon coated, showing a region with extensive horizontal joint-surface-parallel cracks in HAC and SCB filled with HDMI. FW = 360 µm. **d**, **e** 3D 20 kV BSE-SEM macerated preparation of TB Mc3 condyle showing extensive cracking and the extrusion of HDMI to make ridges which are just proud of the ACC MF. FW = 1782 µm in D and 600 µm in E

From our earliest studies with young TB horses in ‘training’, we have been able to demonstrate micro-cracks in ACC in BSE-SEM of PMMA embedded blocks (Figs. 5, 6) [54]. These cracks run mostly parallel with the principal collagen fibre orientation in deep joint cartilage, namely perpendicular to the joint surface. They may be filled with an electron-dense (white in BSE-SEM) material which we call High-Density Mineral Infill (HDMI). Similar cracks form in bone and are similarly filled. We suggest that this evidences a universal crack sealing and healing mechanism—a proposal which contradicts the widely accepted view that cracks in bone are open. Cracks will be open in all the usual, run-of-the-mill standard histological material because decalcification removes HDMI, effectively and entirely. The insignificant ‘organic matrix’ content remains to be isolated and identified. This is one of the most important contributions which needs to be made in the whole field of bone organ biology.

The infilled cracks which develop in vivo need to be distinguished from those which form as an artefact of *post mortem* sample preparation. Despite the rigorous dehydration of tissues with ethanol or acetone prior to substitution with methacrylate monomer during the embedding process, firmly bound structural water is retained in both cartilage and bone matrices. When the polished or micro-milled PMMA block surface is exposed to high vacuum for prolonged periods, as in the SEM specimen chamber, this water can be removed, causing shrinkage and cracking, and particularly in ACC where the proportion of transverse and oblique fibrils is so low in comparison with the bulk of the longitudinal collagen in deep HAC and ACC as not to be able to prevent this occurrence. These artefactual cracks may also develop apparently white edges in BSE imaging due to increased opportunity for electrons to escape from the block surface via the cracks. However, such cracks are themselves empty and appear black, unlike the real HDMI-filled cracks (Figs. 5, 6).

Larger filled ACC cracks contain large amounts of HDMI (Fig. 5c). Some run obliquely, and sometimes they course more nearly parallel with the ACC MF surface (Fig. 6c). Surfaces prepared at a tangent to this surface show that the cracking and filling pattern can be quite complex and is strongly reminiscent of rock cracking and infilling in geology (Fig. 6a).

From our studies of the distal condyles of equine third metacarpal and metatarsal bones (Mc3, Mt 3) and particularly in the Hong Kong POD study material, we can say that, generally speaking, regions with extensively cracked ACC will be indented, sunken or depressed and there will also be damage in the SCB layer. This may take the form of criss-cross cracking of SCB, with the cracks again filled with HDMI (Fig. 5a, c). Damage in the SCB domain will frequently be ‘repaired’ by osteoclastic resorption and new bone deposition. However, during the resorptive phase, the ACC-SCB interface at the osteochondral junction is locally unsupported, and further application of functional load now causes extensive collapse around a developing lesion which can be seen with the naked eye, if the joint surface can be inspected during arthroscopy, or, in our case, on post mortem examination [17–20].

**Fig. 7:**
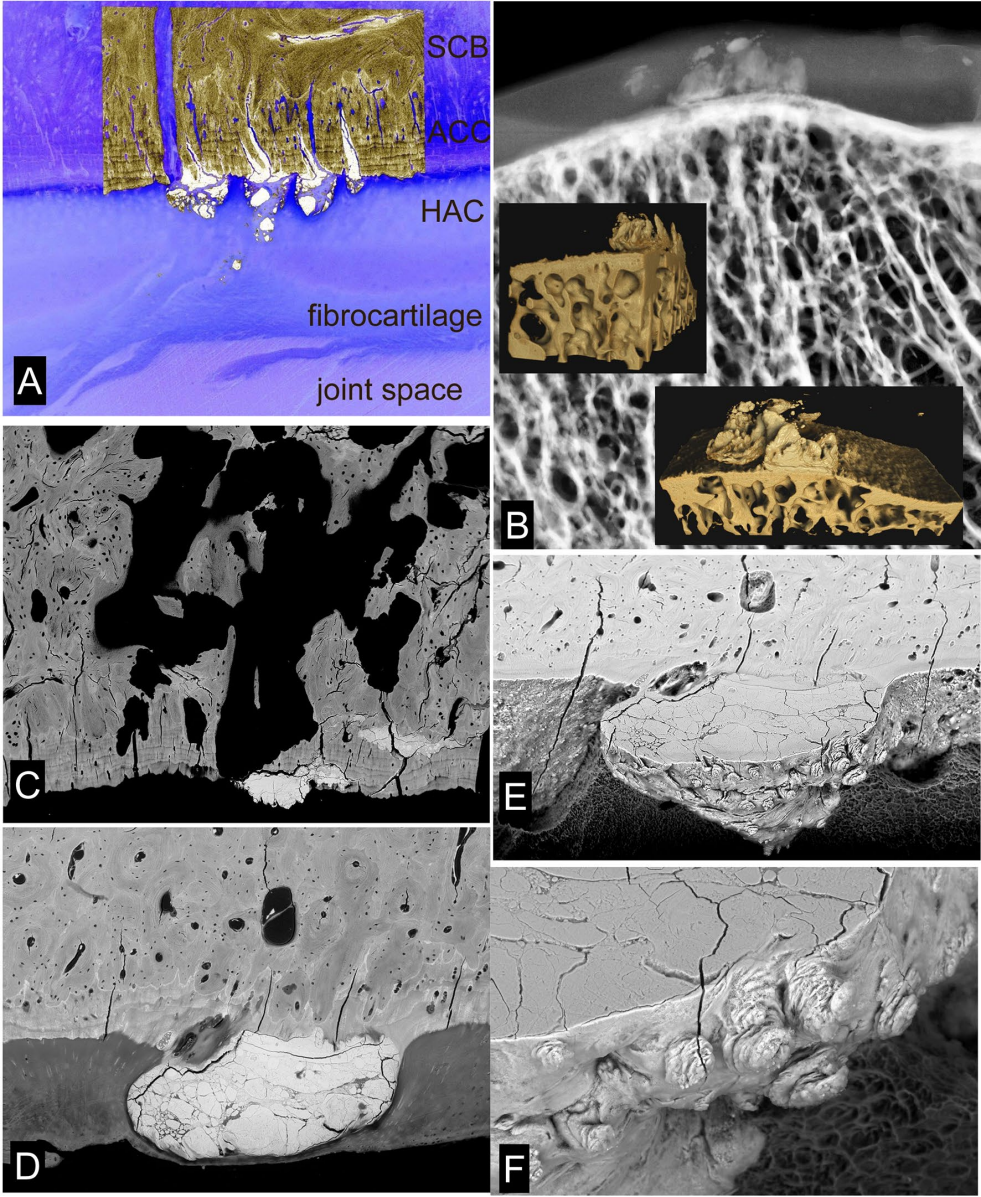
HDMPs in Thoroughbred Mc3, Icelandic horse tarsus and human femur AKU. **a** 20 kV BSE-SEM of TB Mc3 condyle, embedded in PMMA and carbon coated (in top of field), combined with negative of confocal autofluorescence image [the blue cast is the colour negative of yellow green] which shows the full extent of the damaged cartilage in lower part of field. HDMI has projected into HAC as HDMPs which have fragmented. Field width of BSE-SEM component is 900 µm. **b** Fig. 25. HDMPs in human femoral head, AKU case reported in [27]. Faxitron point projection X-ray microradiographic image (Faxitron 26 kV) of ~ 4 mm thick slice showing prominent HDMP complex which extends most of the way through the HAC. Insets are Drishti [ANU Canberra software] reconstructions from XMT data. **c** 20 kV BSE-SEM of TB Mc3 condyle, embedded in PMMA and carbon coated showing HDMI and HDMP with extensive resorption cavity in SCB and ACC. Hong Kong POD study sample B330lfB. FW = 2700 µm. **d** Large HDMP conglomerate in Icelandic horse tarsus (centrodistal joint—see Fig. 8 in [21]) occupying the full thickness of the HAC. 20 kV BSE-SEM after iodine vapour staining to show the HAC. FW = 1555 µm. **e** The same field as in D after plasma ashing to remove PMMA and HAC, tilted at 39°, showing projections from the main HDMP. These are difficult to find in single section planes (polished block surface levels). 20 kV BSE SEM. FW = 1555 µm. **f** Part of field in E at higher magnification. FW = 524 µm

HDMI may not be confined to the calcified tissues but extend outwards from the ACC MF and into the HAC domain to form High-Density Mineral(ized) Protrusions or Protuberances (HDMPs) (Fig. 7). These were first seen in our studies of the Oswestry ‘HOACC’ femoral head project [24]. We also found them in the NZ MUGES TB horse study material [12], in the Hong Kong POD study [18], in AKU-related OA in human femur [27], in Icelandic horse tarsal bones [21, 22], and in Standardbred horse carpal bones [23]. Laverty (personal communication) has recently shown them in horse femoral head HAC.

HDMPs vary from low profile linear extrusions along ACC cracks (Fig. 6d, e) to discrete nearly cylindrical projections (Fig. 7a). The largest may occupy most of the thickness of HAC (Fig. 7b, d). They are all large enough to be seen in XMT (µCT: Fig. 7b insets) and there will probably be the chance of their retro-discovery in many existing data sets [23] when they are looked at more carefully—for morphological detail—rather than used as fodder for blind software calculations.

In re-examining cases with HDMPs I have resorted to ambient temperature plasma ashing which oxidises and removes both the embedding PMMA and all unmineralized matrix components to reveal mineral and mineralizing fronts. This is a gentle approach to making superficially anorganic surfaces for SEM study but is very slow—we achieve rates in the range 20–100 µm per day. Nevertheless, the most convincing demonstration of HDMPs comes with those in block face sections and BSE-SEM imagery. With subsequent plasma ashing, we see both the section profile and the actual surface of the protrusion, which adds a new descriptive dimension. We can clearly see that the larger HDMP have smaller projections upon their advancing surfaces (Fig. 7d–f).

**Fig. 8:**
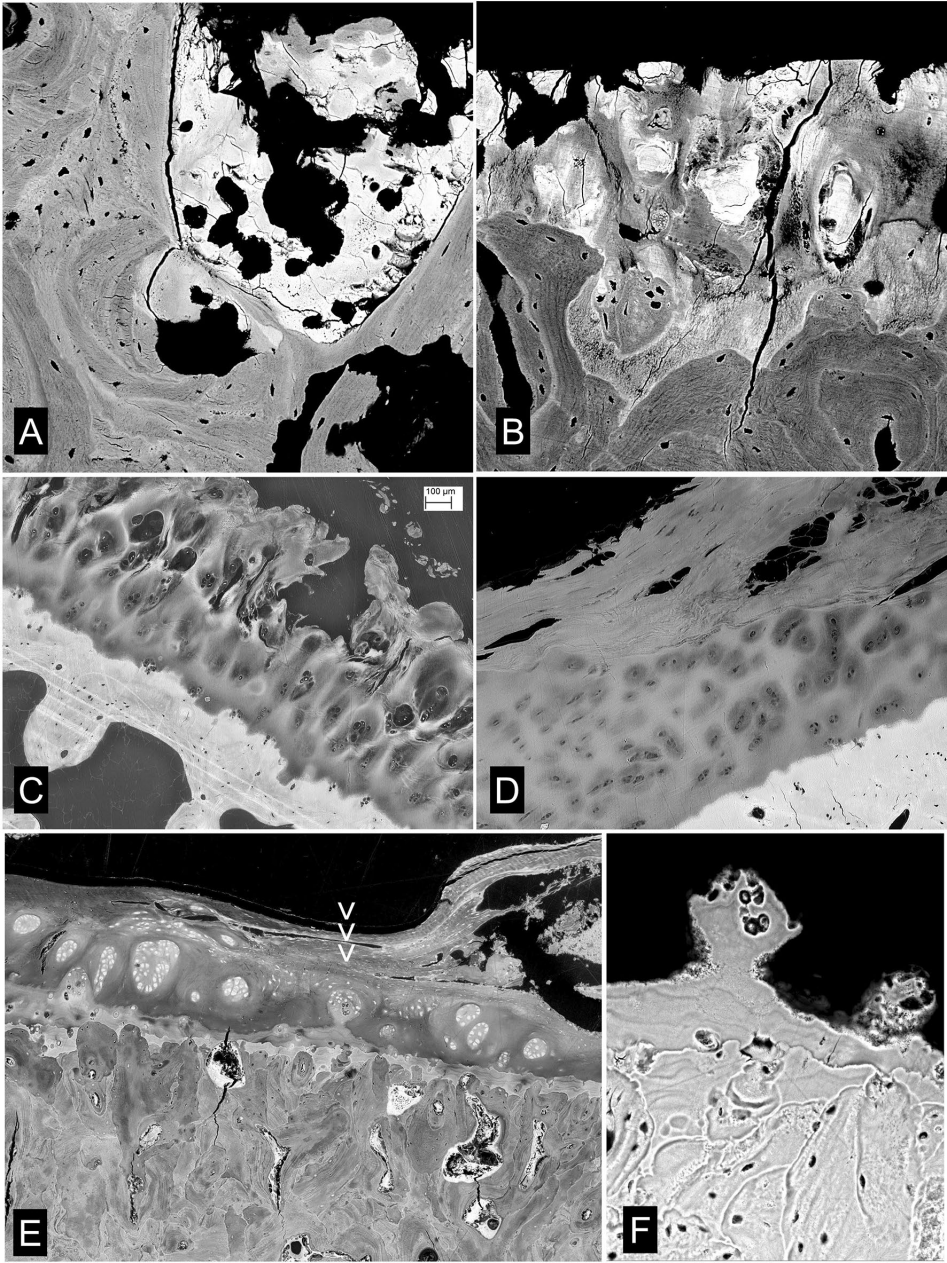
Impacted HDMi/HDMP fragments in human OA, iodine staining histology, chondrocyte clustering/cloning [chondrones]. **a** Human OA femoral head, vertical slice embedded in PMMA, 20 kV BSE-SEM showing dense material impacted in a depression surrounded by bone in an eburnated surface FW = 594 µm. **b** Human OA femoral head, vertical slice embedded in PMMA, 20 kV BSE-SEM showing dense material impacted in calcified cartilage in an eburnated surface (top). FW = 594 µm. **c** Human OA femoral head, vertical slice embedded in PMMA, 20 kV BSE-SEM of micro-milled surface after iodine vapour staining, uncoated, chamber pressure 50 Pa. HAC shows typical features of OA with chondrocyte clustering and fibrillation. The straight parallel lines in the SCB are grooves caused by the micro-milling process. FW = 1833 µm. **d** Same sample, showing region where the surface of the cartilage is fibrocartilage with surface parallel orientation. FW = 1565 µm. **e** Icelandic horse tarsus, proximal surface of T3, showing typical OA changes with chondrocyte clones. PMMA block surface stained with solution of Iodine in Ammonium iodide solution (tri-iodide staining). VVV points to main feature in next image. 20 kV BSE SEM. FW = 2028 µm. **f** Part of same region after plasma ashing to reveal mineralized projections from the ACC MF arising from mineralization in and around chondrocyte clusters. FW = 301 µm

All images of HDMPs show them to be agglomerates of regions which formed from separate mineralization centres, strongly reminiscent of extra-skeletal soft tissue calcification as studied by SEM [55]. Their morphology suggests that they might easily fragment. They are hard and dense [24]. Pieces of similar density may be found impacted into eburnated joint surfaces [24] (Fig. 8a, b). We sometimes see isolated high-density pieces in the HAC in our PMMA blocks, but we cannot prove that we did not move them there during the sample preparation. Nevertheless, it is tempting to suggest that as the hardest and sharpest and densest biological particles to be found in the joint domain that they may be involved in the mechanical degradation of joint cartilage.

HDMPs are not the only projections from the ACC MF. At the finest scale, the MF is most advanced in the immediate vicinity of the chondrocyte lacunae—in their walls—and the highest mineralization densities are also found close to the lacunae. Where the obvious [chondron(e)] cell clusters typical of OA form, the associated advance if the MF is often a prominent feature and one could conceive that the projections might also fragment (Fig. 8e, f). A very important contribution to calcified tissue fragments in the HAC comes from broken ACC with some attached SCB where resorption cavities have undermined the osteochondral junction. These fragments are clearly not an artefact of our interventions in obtaining the tissue and are also candidates as HAC shredders.

Conventional decalcified tissue histopathological reports naturally concentrate on that which can be seen, and therefore emphasise the major HAC changes including matrix histochemical staining parameters, fibrillation and chondron(e) or cell cluster formation. We can, with iodine staining of block surfaces, now also report on these features which were previously invisible with PMMA + BSE-SEM imaging (Fig. 8c, d).

As regards ‘fibrillation’, studies of young and healthy equine joint cartilage and younger and healthier human tissue by freeze fracture and by dry fracture (tearing) after critical point drying methods [33] show that the underlying features which are generally regarded as pathological are there all the time. They are only made the more obvious in OA, and the opinion that ‘fibrillation’ is only pathological may be largely influenced by the fact that pathologists do not elect to study normal tissue.

Once the original HAC histology of surface parallel collagen orientation at the articular surface, with fibre bundles curving down in all directions to be principally normal in the deep layers has been lost, it is never regained, and any reparative ‘fibro-cartilage’ tissue has surface parallel orientation and is more like a dense fibrous connective tissue than cartilage.

If and when the normal HAC-ACC-SCB arrangement is lost, there may be regions where the deep layer of the substitute cartilage undergoes mineralization analogous to the usual situation. Frequently, however, the surrogate cartilage is attached via a layer of bone which has some of the properties of both immature, ‘woven’ bone and Sharpey fibre bone. Many of the collagen fibres in this fibrocartilage [again better to call it dense fibrous connective tissue] insert into this bone of attachment.

**Fig. 9:**
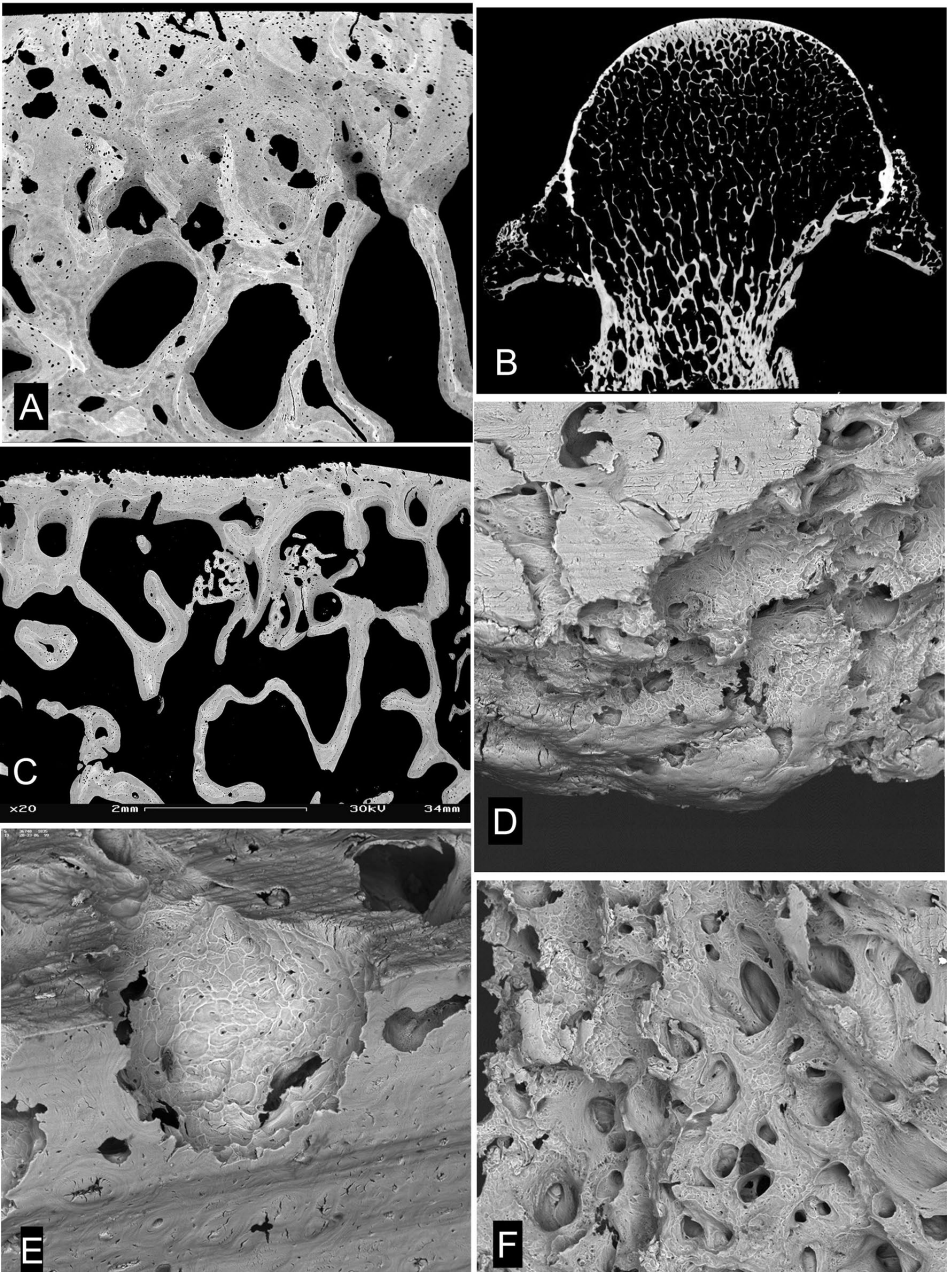
Eburnation and cavitation in human and equine OA. **a** Human femoral head removed at arthroplasty, vertical slice embedded in PMMA, 20 kV qBSE-SEM of micro-milled surface, carbon coated. Articular surface at top is eburnated and SCB is very dense with much prior marrow space filled, mostly with lower density bone. FW = 2700 µm. **b** Three successive 30 µm XMT slices of the whole femoral head and close to the block surface shown in A combined in an RGB composite. A derives from the top dead centre of this image. Note extensive osteophytes at the margins of the head with ‘duplication of the tidemark’: i.e. new HAC with new ACC MF and new SCB. **c** Human femoral head removed at arthroplasty, vertical slice embedded in PMMA, 30 kV BSE-SEM of micro-milled surface, carbon coated. Articular surface at top right is eburnated. Extensive woven bone microcallus has formed in SCB domain. FW = 5750 µm. **d** 20 kV 3D BSE-SEM of macerated Mc3 condyle of Tb horse with large POD lesion. Surface below is eburnated over elevations: at top left is the cut surface of the slice: centre shows an extensive cavity with numerous osteoclastic resorption profiles. FW = 1782 µm. (3D movies of this and similar examples in supplementary information). **e** 20 kV 3D BSE-SEM of macerated Mc3 condyle of Tb horse. Surface below is eburnated and flat. Above is the cut surface of the slice with an extensive cavity with numerous osteoclastic resorption profiles. Is this resorption through an eburnated surface or eburnation of a resorbed surface? FW = 2700 µm. **f** 20 kV 3D BSE-SEM of macerated Mc3 condyle of Tb horse. Part of an extensive cavity with numerous osteoclastic resorption profiles. One spot only (towards top left side) of the surface is eburnated. FW = 1782 µm

**Fig. 10:**
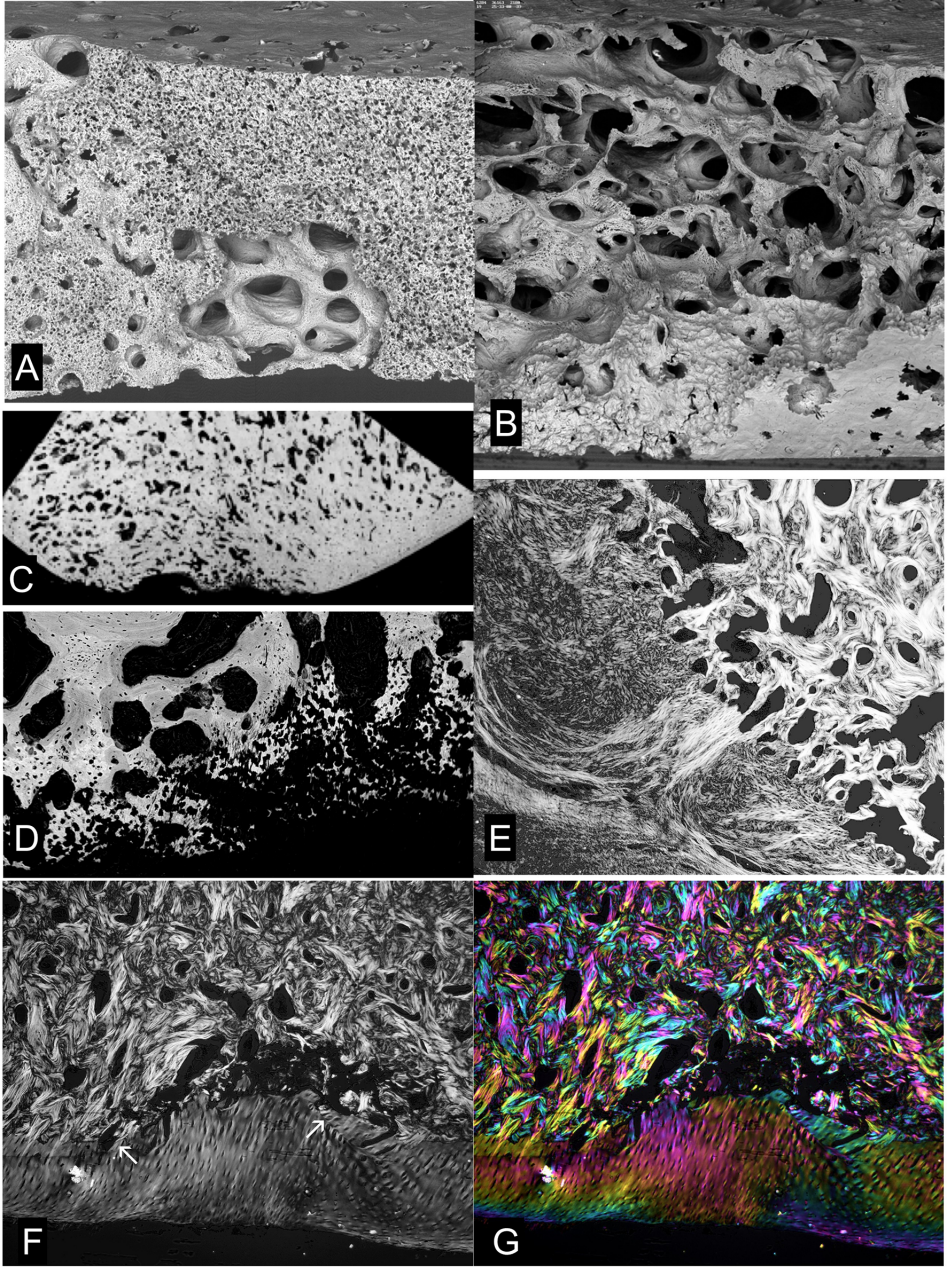
Cavitation and repair in equine OA (POD). **a** An extensive cavity in a POD lesion being repaired with new woven bone formation with numerous closely spaced osteocyte lacunae. 20 kV 3D BSE-SEM of macerated Mc3 condyle of Tb horse. Cut surface of slice at top. FW = 2700 µm. **b** Eburnated surface at bottom right bordering an extensive POD lesion cavity with numerous osteoclastic resorption profiles. 20 kV 3D BSE-SEM of macerated Mc3 condyle. FW = 2700 µm. **c** Low magnification version of 20 kV BSE-SEM montage showing block surface section through entire POD in a slice parallel to that in A. FW = 22 mm. **d** Higher magnification showing the extensive thickness of the layer of woven bone in the base of the deep resorption cavity. FW = 1783 µm. **e** Extensive POD lesion cavity is filled with dense fibrous connective tissue firmly attached to bone. Complex PLM image of 15 µm decalcified section from another parallel slice (to A and C). Six images were recorded with 15° rotations of crossed linearly polarising filters were summed to give an image similar to that produced using circularly polarised light [50]. Brightness is greatest when collagen is lying in the plane of the section. FW = 2800 µm. **f**, **g** TB MC3 case with total loss of ACC and superficial SCB plate at the centre of the POD lesion. ACC is missing entirely in the centre of the field though it can be seen on each side as far as the arrows. Complex polarised light images of 15um decalcified section of TB Mc3 condyle. Six images were recorded at 15° rotations of crossed linearly polarising filters. In the monochrome version, E, the images have been summed: brightness shows the extent to which collagen is parallel to the plane of section. In the coloured image, G, the six images have been assigned the colours Red, Yellow, Green, Cyan, Blue, Magenta in a colour circle sequence composite: colour codes for orientation in plane and again brightness shows the extent to which collagen is parallel to the plane of section [50]. FW = 3050 µm

**Fig. 11:**
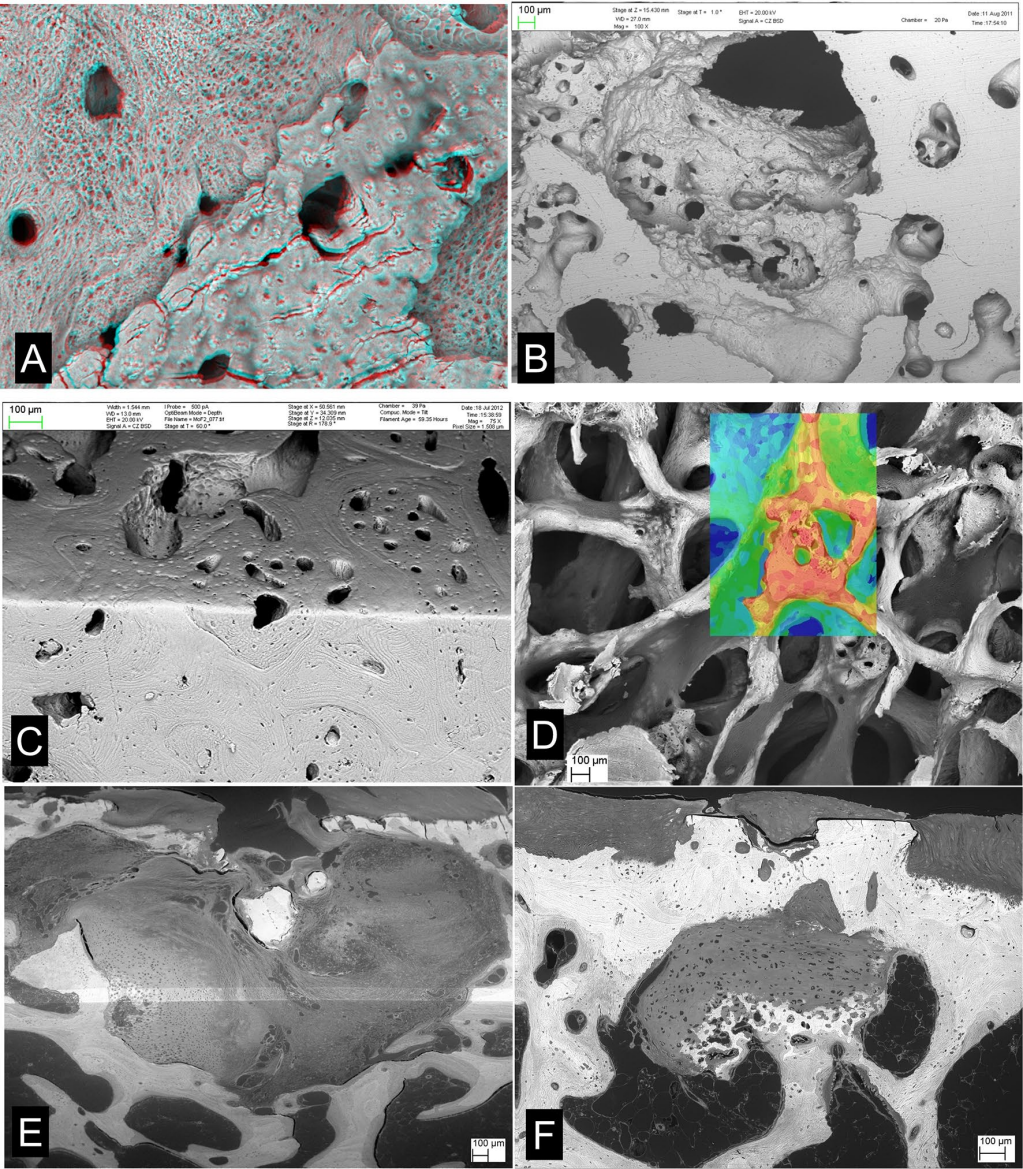
Cavitation and repair in equine and human: BML. **a** 20 kV 3D BSE-SEM anaglyph stereopair image of depressed POD lesion in TB MC3 showing part of the ACC MF at bottom right and woven bone repair in the base of the resorption cavity at top left. FW 900 µm. **b** Macerated femoral head removed at operation for arthroplasty. Cut through sub-articular resorption cavity. 20 Pa chamber pressure, no coating. 20 kV 3D BSE-SEM image, one of an extensive through tilt series. FW = 3600 µm. **c** Same case. Two surfaces at right angles: below the centreline is the naturally eburnated surface: above is a cut made and polished in the laboratory. Both show the extreme volumetric densification of the SCB by infilling with both woven and lamellar bone. 20 kV 3D BSE-SEM image, one of an extensive **through tilt** series. Uncoated, SEM chamber pressure 39 Pa. FW = 1527 µm. **d** Same case. 20 kV 3D BSE-SEM image is one of an extensive **through focus** series. Inset shows 3D contour-level map for this area derived from the through focus image set using AutoMontage software (Syncroscopy, Cambridge, UK: [40]). Most of the trabecular surfaces in this field are covered with non-mineralized osteoid which appears darker than mineralized bone. FW = 3600 µm. **e** 20 kV 3D BSE-SEM image of distal femur removed at operation for arthroplasty, uncoated, SEM chamber pressure 49 Pa. Tissue embedded in PMMA: two block faces prepared at right angles and viewed simultaneously. Ammonium tri-iodide staining to show non-mineralized tissue components as well as the mineralized. Field shows part of a Bone Marrow Lesion identified by pre-operative MRI scanning: note extensive dense fibrous connective tissue marrow and new cartilage. The new cartilage is mineralizing. FW = 3278 µm. **f** Same case as E. ACC surface had been eburnated at top centre and resorbed and cavitated elsewhere, but entire surface is covered with secondary fibrocartilage which also covers the eburnated areas. New cartilage has formed internally which is mineralizing. FW = 2007 µm

It is perfectly obvious in observing material from all the equine distal metapodial condyle overload exercise ‘models’ that we have studied that the most serious changes in ACC, SCB and HAC occur where the impact load on the joint surface is heaviest, i.e. where the palmar/plantar condyles are loaded by the proximal sesamoid bones and it is likewise obvious in the relevant locations in human hip and knee OA (Figs. 8–11). Continued heavy loading leads to continued infilling of potential fatty marrow space with new, immature, woven bone (Fig. 8a, b). Eventually, all the space is occupied, except that necessary for the maintenance of a blood supply. One would imagine that this solidification would reduce the elastic compliance of the SCB bone domain and thus overload the HAC and lead to its degradation. In any event, the HAC is lost locally and bone to bone contact established causing bone wear and eburnation (Figs. 8–11).

Highly polished eburnated areas in human femoral heads show very compact [low space volume fraction] bone at their surfaces in which the fractions or original lamellar-trabecular and space-infilling-immature bone are easily distinguished. The polish on the wear surface is remarkable, and at least equally as good as that which we can achieve in the laboratory with wet, unembedded bone (Fig. 11c).

After a prolonged period of ‘hard work’ in training and racing we would anticipate that the suppression of a normal level of turnover—leading to the reduction in the number of cutting cones attacking the deep surface of the ACC—would be maximal at the same time that the accumulated risk of local injury would be greatest. The horse showing clinical signs of lameness is given a break (‘spelled’). This is the antidote to the stimulus to maintain local bone mass. Catch-up resorption follows. Significant holes develop in the SCB domain. The risk of further injury upon a return to ‘work’ is thus increased. The unsupported ACC + SCB cavitates, probably leaving a depression with rather sharp edges which will put the HAC at risk. Morphological features in equine overload and human OA are remarkably similar. Articular surfaces are left with holes (ulcers) which may be bordered by eburnated zones. The depths of the holes show intense activities of resorption and the deposition of new tissues, including woven bone, dense fibrous connective tissue and newly formed cartilage (Figs. 8–11).

Although it has nothing to do with OA as such, life or career threatening injury in the equine athlete is a further risk incurred due to excessive resorption repair. Incipient breaks in the ACC which spread into the SCB in racehorses can initiate antero-posterior fracture of the condyles, particularly the less massive lateral condyle of the distal Mc3 or Mt3. This risk is heightened by the micro-anatomy of the bony arrangement deep to the SCB [1].

When things get really bad in human hip and knee OA, we have total HAC loss over large parts of the joint surface, and as the consequence of continued eburnation, the ACC is also worn away. We do not see such drastic changes in equine joint samples, probably because our animal rights and welfare considerations would not permit such suffering. The joint responds by elaborating marginal osteophytes in which new bone and cartilage forming tissue is generated: new mineralizing fronts in the new cartilage constitute a genuine reduplication of the tidemark (Fig. 9b). Eventually we have an arthroplasty. The patient’s bony woes do not cease at this point. Whether cemented or cementless, the rigid metal components shield the host bone site from the experience of normal loading and a localised osteoporosis will arise. The placement as such may cause massive death of bone tissue, not least from the exothermic reaction of the setting bone cement. Dead osteocytes (and bone without a covering of osteoblasts) cannot exclude and/or downregulate bone mineralization: this is the main function of bone cells, to prevent overdosing with mineral. The bone becomes hypermineralized and brittle and more likely to fracture.

Before the end stage of joint replacement in human OA, we see more and more woven bone botching-up in bigger resorption cavities and ‘Bone Marrow Lesions’. Kuttapitiya et al. [30] showed that the so-called bone marrow lesions identified by clinical MRI imaging were occupied by uncalcified cartilage and fibrous connective tissues when not by cystic space. The new cartilage is a ‘growth’ cartilage and calcifies [31] (Fig. 11e, f).

The deposition of woven bone in marrow space in overload arthrosis in the horse and in OA in man is similar, morphologically, to the deposition of woven bone in crush fractures of lumbar vertebral bodies [56].

**Fig. 12:**
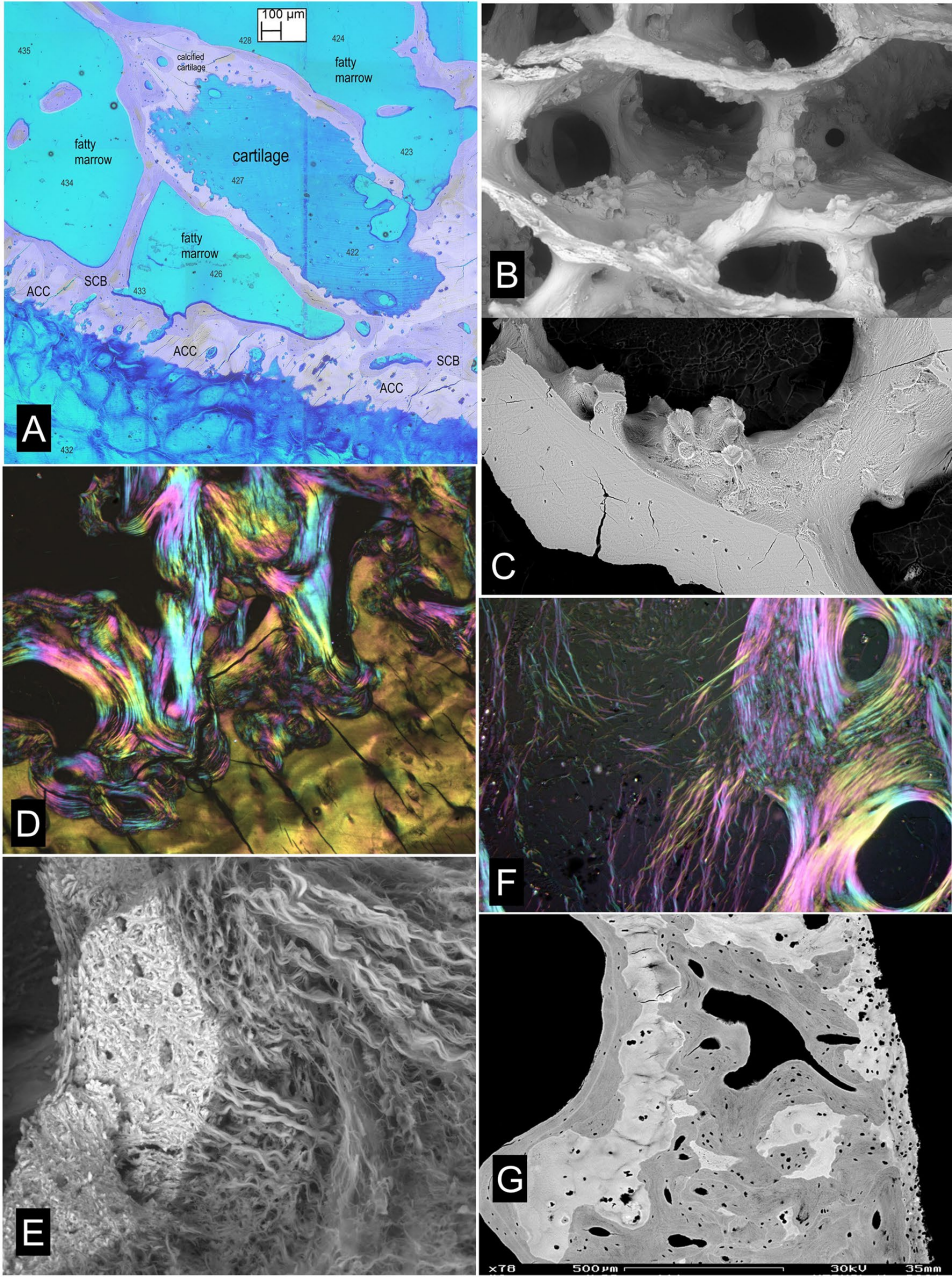
Cartilage in bone, Trabecular excrescences (adipocytic bone moulding) Sharpey fibres in trabeculae, tidemark reduplication. **a** 10um section prepared by Laser Ablation Microtomy [48] of PMMA block of distal femur removed at operation for arthroplasty. The light microscopy slide was imaged by 20 kV 3D BSE-SEM (grey components; uncoated, SEM chamber pressure 50 Pa) and transmitted light microscopy after MacNeal’s tetrachrome staining (blue components). The images were combined using ‘Honza’s programme [43]. A ‘trabecula’ is composed of hyaline cartilage at its centre which is mineralizing peripherally. This would appear as a small BML in clinical MRI. FW 7367 µm. **b** Trabecular excrescences. 20 kV 3D BSE-SEM of subchondral trabecular bone in a macerated OA knee sample. FW = 3157 µm. **c** Trabecular excrescences. 20 kV 3D BSE-SEM of subchondral trabecular bone in an embedded, micro-milled OA femoral head sample: PMMA has been removed to some depth by plasma ashing. The original block surface domains are flat. FW = 1208 µm. **d** (Same case as 12a). Human distal femur, osteochondral junction, unstained, undecalcified 10 µm Laser Ablation Machined section from osteoarthritis case (knee arthroplasty), complex polarised light image [49: & see caption to Fig. 10g]. A trabecular excrescence is seen in top left of field, ACC in bottom right. FW = 1220 µm. **e** 20 kV BSE-SEM of iodine vapour stained, fractured PMMA embedded trabecular bone from OA knee case, showing dense fibrous marrow in subchondral bone with fibres inserting into bone trabecula. FW = 412 µm. **f** Severe OsteoArthritis in Alkaptonuria femoral head. 8 µm Decalcified unstained section kindly loaned by J.A.Gallagher. Complex PLM [49: & see caption to Fig. 10g]. Sharpey fibre bundles run from marrow into bone trabeculae. FW = 605 µm. **g** Human femoral head, vertical section embedded in PMMA, micromilled, carbon coated, 20 kV BSE-SEM, showing ‘duplication of the tidemark’. The old layer of ACC is embedded in SCB at the left of the field. A new mineralizing front is seen at right. FW = 1488 µm

Our SEM and PLM studies show that an extensive development of fibrous marrow which replaces and displaces the original fatty marrow is also a typical change in OA. Very remarkably, Sharpey fibres are inserted in directly into trabeculae deep to any external bone surface in OA samples (Figs. 11e, f, 12e, f).

Trabecular excrescences (Fig. 12b–d) were originally discovered in AKU-OA [26] and it was only later realised that these are a normal feature of usual OA in humans. Whether they are part of a normal age change, they are certainly have been present in each OA case where we have looked for them, but then of course all our OA cases have reached a certain age. As to their functional significance, we do not know. They are certainly moulded by adipocytes, but it seems improbable that their bone matrix is made by other than osteoblasts. They may be added on to any trabecular surface, i.e. whether, as in the majority of cases, fully mineralized and previously resting, or whether previously resorbed. If they are located near previously resorbed (resting after resorption) areas, they may not cover the entire resorbed territory. The bone of which they are formed may be slightly more highly mineralized than the bone to which they attach [26]. The matrix is lamellar (Fig. 12d). They are none too obvious in ordinary decalcified section histology, but the aware observer will see them, and it is surprising that they were not spotted previously. They are outstandingly obvious in 3D SEM images (Figs. 12b, c).

## Discussion

The tough, rubbery hyaline articular cartilage (HAC) and the subchondral bone (SCB) in a diarthrodial joints are united via the medium of the layer of articular calcified cartilage (ACC) which is seemingly none other than HAC in which a substantial fraction of its large water content is replaced by carbonated-hydroxapatite mineral. This stiffens the ACC and provides a rough modulus match to mineralized bone tissue, but the composition and the orientation of the collagenous matrices of the deep layers of HAC and bone are profoundly different. In particular, most of the Type 2 collagen in cartilage is normal to the joint surface, whereas the Type 1 collagen in bone matrix has many orientations, all parallel with the formative surface in mature bone, but complicated by the crossed lamellar organisation controlled by osteoblastic movement during its secretion. Although the mineral concentration and the nanomechanical properties are correlated in both ACC and SCB, the correlations are different in the two tissue types [11, 13, 24, 57].

The union of HAC to ACC is assured because they are one and the same tissue originally: the protein-polysaccharide structure is continuous. Water is replaced by mineral, but there is an accompanying subtle change in matrix chemistry which enables us to see, by staining, which part had been calcified after the tissue has been decalcified according to standard histopathological processing schedules.

The union of SCB to ACC is maintained by piecemeal osteoclastic ‘cutting cone’ resorption of the deep layer of ACC, with osteoid deposition upon the locally pocked bone surface, its mineralization, and reunion. Thus the ACC-SCB junction is normally maintained by regular replacement in tiny, discrete regions, where only a bit is patched up or replaced at a time. Nevertheless, ACC is used up—expended—in the process, and unless the mineralizing front (MF) of the ACC advances correspondingly to increase its thickness, the ACC would disappear. This is of no concern if the animal is growing, because expansion of the thickness of HAC continues after the closure of the growth plate in a long bone. After this element of growth has ceased, however, the expansion of the HAC is the only growth mechanism left, and when this stops, it is a matter of time before HAC thickness is reduced by the advance of the ACC MF. If a certain thickness of HAC is ideal, then the situation falls short of an ideal.

Multiple tidemarks are normal, as is layering in the degree of mineralization, and especially whilst the individual is growing. The density layering in ACC can be compared with an earthquake bearing. This absolutely normal layering should not to be confused with the pathological ‘duplication of the tidemark’ that is a hallmark of advanced OA changes and would better be called’(re-)institution of a new mineralization process in secondary repair cartilage’.

It is very well known and understood that the amount of mineral within a microscopic volume of bone matrix increases with time. Immature, woven bone usually mineralizes much more rapidly than mature lamellar bone, which shows a prolonged and distinct maturation phase. The most important function of ex-osteoblasts whether as surface cells or osteocytes, is to hold this process back, to regulate mineralization by down regulation, to prevent bone from becoming too stiff and brittle.

Does ACC also have a maturation phase? We do not know for sure, but why not? There is plenty of water-filled space left to be filled with mineral in less mature cartilage. ACC has a wide range of degrees of mineralization with concentrations varying from below that of any normal bone to those which are much higher. Levels are generally higher in mature human ACC than in the neighbouring SCB. In studies in younger horses with calcification-front-seeking labels like tetracycline and calcein, a clear correlation of labelling lines with the advancing front was found [8–10]. Equally, we could show the general uptake of labels in all less mature bone, meaning that labels are also bound into bone undergoing maturation, and not just at the mineralizing surface. We know of one report in the literature which found a general distribution of single tetracycline doses to several ‘tidemarks’, i.e. to ACC in bulk, in human OA [58]. This would be expected if ACC undergoes maturation. If it does, and if turnover at the osteochondral junction is reduced with age (as certainly seems to be the case from my reading of the runes), then HAC is attached to SCB by *thinner* layers of *denser* ACC and this alone is a risk for fragility at this critical junction, and yet another factor in ageing and OA.

As microscopists, we are careful to distinguish fabric density—mineral concentration at the micro scale—from any other meaning of density and remember that such distinctions are completely lost and irrecoverable from clinical imaging modalities. If we say that bone ‘becomes denser with exercise’, we must mean that more bone is packed into the available space. The new bone added is less dense. We just have more of it, and this occurs by the deposition of new bone in prior fatty marrow space remote from contact with local bone as well as by thickening of existing ‘trabecular’ structures. The latter become so robust that new systems of intra-trabecular blood vessel canals are established to maintain the vitality of the bulkier bone baulks.

Invasion into HAC by osteoclasts in the mature individual leads to the loss of the area of attachment of cartilage to bone and it is another part of the whole complex of changes in OA. Cathepsin K is a major proteolytic enzyme used by osteoclasts to destroy demineralized bone matrix [59]. It is also made by chondrocytes in the HAC and in increased proportion in OA [60, 61].

Studying the equine athlete model has allowed us to document the suppression of cutting cone activity (replacement maintenance) by exercise and, vice versa, catch up resorption during spells of rest. This constitutes a particular risk in the development of overload, traumatic OA, because the osteochondral junction is undermined as the result,and therefore more liable to mechanical injury, including local microfracture in the ACC-SCB plate.

HDMI is another feature of OA. Although brought to light by BSE-SEM [54], hypermineralized cracks had been previously identified in human OA ACC using microradiography [62]. The better resolution of SEM showed how micro-cracks seal and heal by the deposition of HDMI [54]. Comparison of femoral heads removed for joint replacement with postmortem control samples showed hyperdense material impacted into in ACC and superficial SCB in OA [24].

HDMI also forms within subchondral bone and may extend beyond the local boundary of the 'tidemark' ACC mineralizing front into the domain of HAC to form HDMPs [12, 18, 21–23]. That they were so long in being flagged up as a factor in OA probably depends on the facts that (1) the huge majority of all histopathological studies of hard tissues use demineralization as the first step; (2) that the HDMI material leaves little residual matrix; and (3) that microradiography was never very extensively used and that it went out of fashion—even though digital point projection X-ray microscopy as in the Faxitron system is extremely easy to use and is very good for prospecting for HDMPs. Cracks and splits as such are seen in demineralized tissue histology [63], but it is difficult to ascertain whether they were present before decalcification and microtomy. Several abstracts from the Gallagher–Ranganath–Thomas–Jeffery group in Liverpool have reported finding HDMPs by MRI clinical imaging in human joints [64]. I feel that we have hardly seen the tip of this iceberg. HDMPs seem to be distinct entities from the central osteophytes which can be found with radiographic and MRI clinical imaging [65–67]. These seem to be bone and calcified cartilage [67].

3D imaging methods, both SEM and XMT based, show clear cut resorption bays (Howship’s lacunae, resorption pit complexes) in eburnated surfaces. It is not always clear as to the order of events. Perhaps eburnation of surfaces with resorbed pits is easier to accept, but resorption through eburnated surfaces is also possible. Surprisingly, we have found instances of the formation of replacement fibrocartilage over an apparently very flat eburnated prominence, but it was clear that this was not well attached (Fig. 11f). For surrogate, substitute cartilage to attach properly, the eburnated surface must be resorbed, when new woven bone can attach inserting fibres and hold the structures together and this does occur wholesale (Fig. 10).

Bone marrow lesions (earlier, bone oedema [68]) are features identified in MRI imaging. Opinions differ as to what histology is to be found including a preponderance of high volume fraction, poorly mineralized bone [69] and regions of enhanced angiogenesis and increased bone turnover [70]. These are not necessarily exclusive. We would also include cysts as such, regions with more or less dense fibrous connective tissue (fibrous marrow) [30] and unmineralized cartilage and woven bone matrix (osteoid) (Figs. 11e, f, 12e, f) [31]. Marrow fibrosis may be a general change in ‘inflammatory and degenerative’ disorders in SCB [71]. We should also note the general similarities of the marrow responses found in normal overload densification, OA, crush fractures and surgical iatrogenic injuries.

The significance of Sharpey fibres inserting into trabecular bone in OA needs to be explained. The usual interpretation of these penetrating, extrinsic fibres is that they transmit load to bone: they normally only form at external surfaces where tendon, ligament of aponeurosis fibres insert. OA bone is therefore abnormal in respect of its trabecular component being loaded from within. Is this seen elsewhere? Yes. Similar fibrous marrow with Sharpey fibres inserting into trabecular components is seen at dental implant sites [72].

Key findings from our studies of HAC, ACC and SCB in the context of OA would be that we risk losing 50% of the story when we throw away 50% of the sample, down the drain, with the demineralization fluid [73–75]. Obviously, we shall continue to make and to stain study decalcified tissues. However, we would also recommend the extended use of whole samples or blocks against sections, and being careful to preserve the mineral phase as part of the normal state of the art for *research* histopathological studies of joints.

Of mice and men? Some of the findings shown here might be duplicated in the smallest experimental mammals, and we cannot do fast gene experiments [47, 76] in man and horse. But let us not think that we can do everything with mice.

## Supplementary Information

Below is the link to the electronic supplementary material.

Supplementary file1 (PPTX 154260 kb)

Supplementary file2 (DOCX 25 kb)

## Data Availability

Supplementary data are available on the Journal site. Image files can be obtained from the author for educational and research purposes.

## Abbreviations

ACC: Articular calcified cartilage
AKU: Alkaptonuria
BSE: Backscattered electron
CSLM: Confocal scanning light microscopy
HDMI: High-Density Mineral Infill
HDMPs: High-Density Mineral Protrusions or Protuberances
LAM: Laser Ablation Microtomy
OA: Osteoarthritis
PMMA: Poly-Methyl-Methacrylate
SCB: Subchondral bone
SEM: Scanning electron microscopy or microscope
TB: Thoroughbred racehorse
XMT: X-ray MicroTomography—the term in use at QMUL and equivalent to µCT

## Code Availability

Custom software for co-registration of images from different types of microscopy as described in [43] in the MS whch is available free of charge from the author.
