# Supplementary material for: The Bone Cartilage Interface and Osteoarthritis
Source: Calcif Tissue Int. 2021 Jun 4;109(3):303–28. doi: 10.1007/s00223-021-00866-9 (PMC8403126; doi:10.1007/s00223-021-00866-9)
Supplement: Supplementary file 2 — Supplementary file2 (DOCX 25 kb) [file 223_2021_866_MOESM2_ESM.docx]

**CTIN-S-21-00169**

**Author: A Boyde**

**The supplementary images** in the PPTX presentation have been named in accordance with the scheme adopted for the illustrations in the main paper. Suppl 1Aa refers to the topic addressed by Figure 1A. Suppl 1Ab to the same topic, in another image showing different insights etc. The movie sequences will play automatically.

**Captions to illustrations. Common abbreviations, reminder.**

ACC articular calcified cartilage

BSE backscattered electron

BV blood vessel usually in the context of BV canal

FW Field width

HAC hyaline articular cartilage

Mc3 Third metacarpal bone, equine, usually the distal condyles

MF mineralizing front [current ‘tidemark’]

OA OsteoArthritis

POD Palmar osteochondral disease (traumatic OA in the horse)

SCB subchondral bone

SEM scanning electron microscopy or microscope

TB Thoroughbred racehorse

**Suppl. 1Aa**. Another example of 20kV BSE SEM image of carbon coated, micromilled PMMA embedded block surface of distal condyle of 2 year old Thoroughbred (TB) racehorse third metacarpal bone (Mc3). HAC (top left) hyaline articular cartilage region which here shows black as it contains no mineral. ACC shows incremental mineralization-front-progress layers with more and less mineral content. It appears that there is zero or a very low mineral content after each growth arrest line (tidemark). SCB (lower right segment) is attached via a hypermineralized reversal cement line to the deep layer of the ACC. after local ‘cutting cone’ resorption events. FW = 1166µm.

**Suppl. 1Ab**. 20kV BSE SEM, ‘BioOss’ bone augmentation material used for improving dental implant survival, from an implant site trephine core sample. This material is made by calcining bovine bone. The presence of ACC and SCB proves that not only trabecular bone is used in the manufacture. The presence of the dark inter-layer lines shows the near absence of mineral.

**Suppl. 1Ac**. 20kV BSE SEM of TB Mc3 macerated in NaOCl bleach to remove non-mineralized components. The ACC is splitting where there was no mineral in the inter-layers between successive ‘tidemarks’. FW = 4450µm.

**Suppl. 1Ad.** 20kV BSE SEM of TB Mc3 macerated, another animal. The ACC has split to reveal the current ACC MF at right and the next deeper ‘tidemark’ at left. Details of the shape of the successive ‘tidemarks’ is identical. FW = 4450µm.

**Suppl. 1B.** Co-registration of BSE-SEM and CSLM images seen in GIF movie format, courtesy of Michael Doube [9, 10].

**Suppl. 1C**. 2 year old TB MC3 from the Bristol Treadmill training experiment, treadmill group, embedded in PMMA+IEMA (iodinated methacrylate). Iodine here gives negative staining contrast. Another example matching Figure 01C. Region shows lees loaded dorsal part of condyle.

.

**Suppl. 1D**. GIF movie version of Figure 01D made by recording the same field of view at multiple tilt angles. 14 frames. To view this using ImageJ, go to Stacks/Animation/Options/ and choose ‘to and fro’ and frame speed 10fps. 20kV BSE SEM image of gold coated PMMA cast of mid-shaft transverse section of 2 yr old TB Mc3. Near the centre can be seen a ‘cutting cone’ coming towards you. FW = 1937µm.

**Suppl. 1Ea**. Same image as Figure 01E, untrimmed, higher resolution. 20kV BSE SEM image of gold coated PMMA cast of marrow space at the osteo-chondral junction in a 2 year old TB Mc3 condyle, made by dissolving embedded bone with sequential acid and bleach treatments and ultrasonic dispersal of osteocyte lacunar and canalicular casts. Many ‘cutting cone’ replicas are seen, large ones with detail of the individual resorption pits by osteoclasts contrast with narrow projections which show the space in shut-down ‘osteones’. FW = 1900µm.

**Suppl. 1Eb.** Adjacent field**.** In this colour version, colour shows the direction of collection of the BSE signal [39,40], i.e., colour codes for the direction of illumination by analogy with ordinary light. FW = 1900µm.

**Suppl. 2A.** GIF movie version of Fig. 2A showing the BSE-SEM and CSLM autofluorescence components separately.

**Suppl. 2B.** Macerated sample. HAC invasion by cutting cones. ACC MF in 6 week old foal Mc3, showing the radically different nature of the MF during rapid growth, which is much more affine to conditions in the growth plate cartilage. Two ‘canals’ penetrate the ACC and it is possible that they were occupied by one capillary blood vessel loop. A thin shell of bone has formed lining the right hand canal. FW = 900µm.

**Suppl. 2CD**. Same field as Figure 2C,D. 20kV BSE SEM images of vertical section through superior surface ACC MF of femoral head of 33 year old male, GIF movie version to show 3D.The same field was imaged at multiple tilt angle differences. FW 2700µm.

**Suppl. 2EF.** Same field as Figure 2EF. Superior surface of femoral head of 75 year old male, MF ACC. GIF movie version to show 3D.The same field was imaged at multiple 2° tilt angle differences. FW 2700µm.

**Suppl. 3D**. Densification by forming new bone in prior marrow space in SCB in human OA femoral head. Quantitative BSE SEM image, same field as Figure 3D. Pseudocoloured version of Figure 3D using ImageJ 16cols LUT. FW = 1350µm.

**Suppl. 3E.** 20kV BSE SEM, femoral head of a PM reference case. Pseudocoloured version of Figure 3E using ImageJ 16cols LUT. FW = 1782 µm.

**Suppl. 3F**. 20kV BSE SEM showing deep subchondral trabecular bone in an OA (elective arthroplasty) femoral head. Differences in the fabric density of the old and the repair bone are better seen in this pseudocoloured version of Figure 3F. ImageJ 16cols LUT. FW = 1114µm.

**Suppl. 7A**. HDMP in human patella OA. Macerated sample showing ACC with HDMP at centre. 20kV BSE SEM.

**Suppl. 9A**. Human OA. Articular surface at top is eburnated and SCB is dense with much prior marrow space filled. Pseudocolour version of Figure 09A, ImageJ 16cols LUT . FW =2700µm.

**Suppl. 9Da.** 20kV 3D BSE-SEM of macerated Mc3 condyle of Tb horse with large POD lesion. Anaglyph stereo-pair version for viewing with Red (for left eye) and Cyan filter spectacles. Tilt angle difference = 6°. Same field as Figure 09D rotated 90° for stereo viewing. Surface at left is eburnated over elevations: at right is the cut surface of the slice: Showing extensive cavity with numerous osteoclastic resorption profiles. FW = 1782µm.

**Suppl. 9Db**. Same field as GIF movie file with 3 views at 6° tilt angle differences. FW = 1782µm.

**Suppl. 9Ea**. Same field as Figure 9E as an anaglyph stereo-pair, tilt angle difference = 6°. Field rotated 90°. POD lesion in TB MC3 condyle. Surface right is eburnated and flat. Left is the cut surface of the slice with an extensive cavity with numerous osteoclastic resorption profiles. FW = 2700µm.

**Suppl. 9Eb**. Same field as Figure 9E and Suppl 09Ea as a GIF movie. Eburnated surface below, cut surface top. FW = 2700µm.

**Suppl. 9Fa**. Macerated Mc3 condyle of Tb horse. Part of an extensive POD lesion cavity with numerous osteoclastic resorption profiles. One spot only (towards top left side) of the surface is eburnated. Same field as Figure 09F. FW = 1782µm.

**Suppl. 9Fb**. Same field as GIF movie file with 3 views at 6° tilt angle differences. FW = 1782µm.

**Suppl. 10A**. Higher magnification view of part of Fig 10A. Cavitation and repair in equine OA (POD). Base of extensive cavity in a POD lesion being repaired with new woven bone formation towards top and right showing the numerous and large, closely spaced osteocyte lacunae. Higher magnification view of Fig 10A. FW 900µm.

**Suppl. 10C**. Higher magnification version of 20kV BSE SEM montage in Figure 10C. FW = 22mm.

**Suppl. 10E**. Colour version of Figure 10E. Extensive POD lesion cavity filled with dense fibrous connective tissue firmly attached to bone. Six images were recorded with 15° rotations of crossed linearly polarising filters used as Red, Yellow, Green, Cyan. Magenta in colour circle sequence. Colour codes for orientation of collagen in plane [49]. Brightness is greatest when collagen is lying in the plane of the section. FW = 2800µm.

**Suppl. 10F**. TB MC3 POD lesion. ACC is missing entirely in the centre of the field. Six images were recorded at 15° rotations of crossed linearly polarizing filters here shown as GIF movie [49]. FW = 3050µm.

**Suppl. 11B.**  Macerated femoral head removed at operation for arthroplasty. Cut through sub-articular resorption cavity 20Pa chamber pressure, no coating. 20kV 3D BSE-SEM image. Same field as Figure 11B. Through tilt series of 28 frames. FW = 2800µm.

**Suppl. 11C**. Same case, same field as Figure 11C. Through tilt series as a 3D GIF file. Two surfaces at right angles: below the centreline is the naturally eburnated surface: above is a cut made and polished in the laboratory. Both show the extreme volumetric densification of the SCB by in-filling with both woven and lamellar bone.. FW = 1527µm.

**Suppl. 11Da**. Same case. Same field as Figure 11D. Through focus series here showing images at 100µm mechanical focus level differences [39]. Many trabecular surfaces are covered with non-mineralized osteoid seams. FW = 2917µm.

**Suppl. 11Db**. Co-morbidity of OA with osteomalacia in equine OA. MC3 POD lesion, for comparison with Figure 10D which showed extensive osteoid which appears darker than mineralized bone. 20kV BSE SEM. FW = 3570µm. Fragments of tissue in marrow spaces result from cutting this 600µm section before embedding.

**Suppl. 12A**. Cartilage in bone trabecula in human distal femur OA. 10µm section prepared by Laser Ablation Microtomy [47]. Same field as figure 12A. Sequence showing LM after MacNeal’s tetrachrome staining and BSE-SEM and mixtures of the two imaging modalities. A ‘trabecula’ has hyaline cartilage at its centre which is mineralizing peripherally.

**Suppl. 12Da**. (Same case as 12A and Suppl. 2Ab). Human distal femur, osteochondral junction, unstained, undecalcified 10µm Laser Ablation Machined section from osteoarthritis case (knee arthroplasty), complex polarised light image [49]. A trabecular excrescence is seen in top left of field, ACC in bottom right. FW = 1220µm. This is a higher resolution copy of Figure 12D.

**Suppl. 12Db.** Trabecular excrescences in the same case (see also Figure 12D). Another Laser Ablation Microtomy section. Level of the field is one field height above the SCB-HAC junction. Sequence shows 20kV 3D BSE SEM; pseudocoloured BSE-SEM using our in-house topo9c LUT; polarised light microscopy and LM after MacNeal’s tetrachrome staining. FW = 1620µm.
